# Supplementary material for: Simultaneously discovering the fate and biochemical effects of pharmaceuticals through untargeted metabolomics
Source: Nat Commun. 2023 Aug 3;14:4653. doi: 10.1038/s41467-023-40333-7 (PMC10400635; doi:10.1038/s41467-023-40333-7)
Supplement: Supplementary file 1 — Supplementary Information [file 41467_2023_40333_MOESM1_ESM.pdf]

Supplementary Information

Simultaneously discovering the fate and biochemical effects of pharmaceuticals through untargeted metabolomics

Tara J. Bowen[1], Andrew D. Southam[1,2], Andrew R. Hall[3], Ralf J.M. Weber[1,2], Gavin R. Lloyd[2], Ruth Macdonald[4], Amanda Wilson[5], Amy Pointon[3], Mark R. Viant[1,2,*]

[1] School of Biosciences, University of Birmingham, Edgbaston, Birmingham B15 2TT, UK

[2] Phenome Centre Birmingham, University of Birmingham, Edgbaston, Birmingham B15 2TT, UK

[3] Safety Sciences, Clinical Pharmacology and Safety Sciences, BioPharmaceuticals R&D, AstraZeneca, Cambridge, UK

[4] Animal Sciences and Technology, Clinical Pharmacology and Safety Sciences, BioPharmaceuticals R&D, AstraZeneca, Cambridge, UK

[5] Integrated Bioanalysis, Clinical Pharmacology and Safety Sciences, BioPharmaceuticals R&D, AstraZeneca, Cambridge, UK

*Correspondence: m.viant@bham.ac.uk

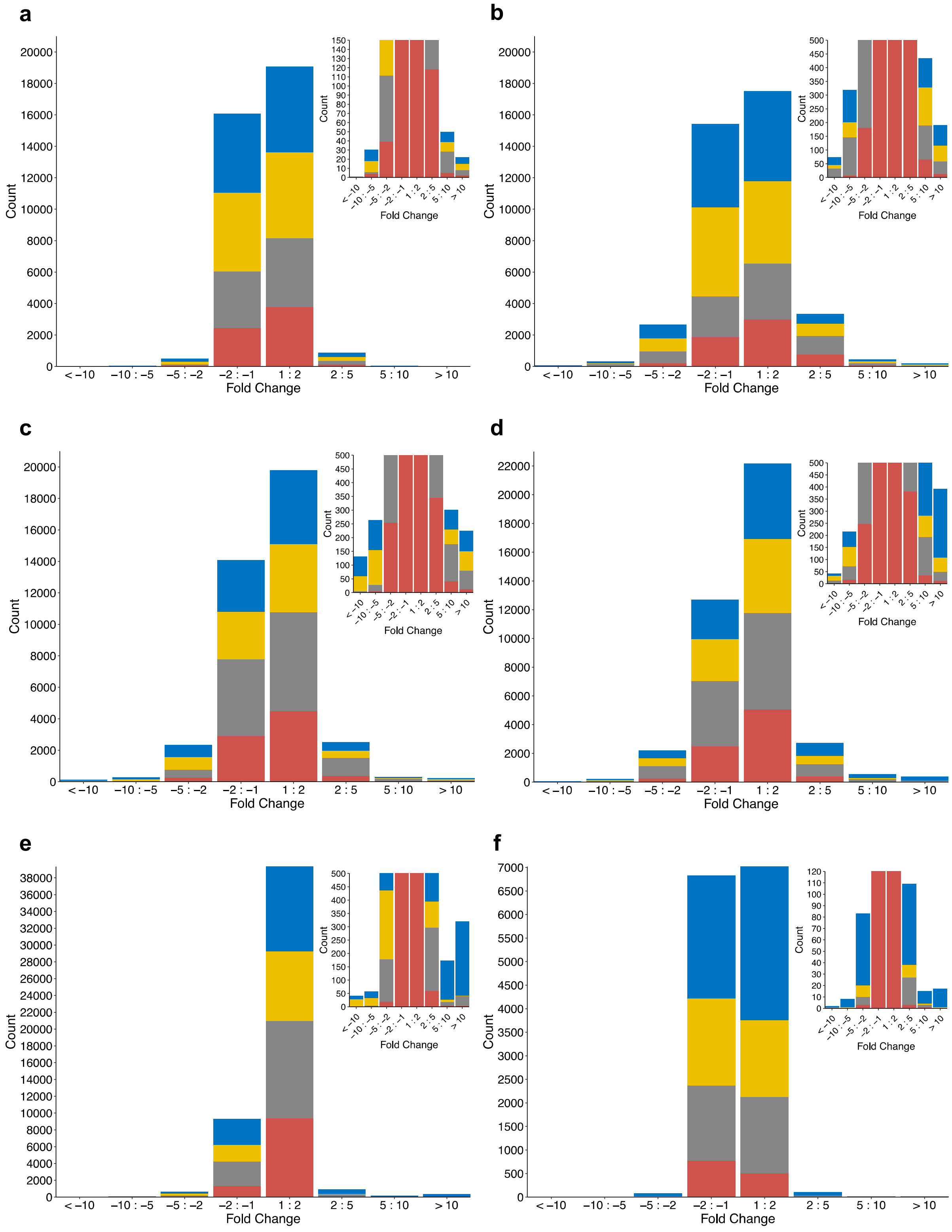

Supplementary Figure 1 Distribution of peak intensity fold changes observed in untargeted metabolomics toxicity datasets. *The distribution of fold changes (median exposed peak intensity compared to median biological control peak intensity per feature) observed in the untargeted metabolomics toxicity datasets measured by HILIC UHPLC-MS in positive ion mode (blue), HILIC UHPLC-MS in negative ion mode (yellow), RP C_{18} UHPLC-MS in positive ion mode (grey), and RP*

C_{18} UHPLC in negative ion mode (red) discussed in this paper: ***a*** *plasma from rats exposed to sunitinib (n = 29),* ***b*** *plasma from rats exposed to KU60648 (n = 19),* ***c*** *cardiac tissue of rats exposed to sunitinib (n = 5),* ***d*** *cardiac tissue of rats exposed to KU60648 (n = 5),* ***e*** *intracellular extracts of hiPSC-CMs exposed to sunitinib (n = 9), and* ***f*** *culture medium of hiPSC-CMs exposed to sunitinib (n = 9). Inset figures display the chart zoomed-in on y-axis. At least 98% of fold changes are below the 10-fold threshold across the 6 datasets.*

Supplementary Table 1 A summary of the datasets analysed*. For each dataset analysed, the experimental model and matrix, exposure (parent) substance, dose(s), measured time points, and the reason for analysis are reported.*

Dataset	Biological system and sample type	Exposure substance	Dose(s)	Sampling time points	Reason for analysis
Sunitinib Rat Plasma	Rat – Plasma	Sunitinib	25 mg/kg/day for 14 consecutive days	4 hrs post-dose on days 1, 2, 4 and 8; 28 hrs post-dose on day 14.	Biotransformation map; temporal tracking of parent and biotransformation product levels; endogenous metabolic and lipid signature
Sunitinib Rat Cardiac Tissue	Rat – Cardiac Tissue			28 hrs post day 14 dose (i.e., day 15)	Biotransformation map; endogenous metabolic and lipid signature
KU60648 Rat Plasma	Rat - Plasma	KU60648	150 mg/kg/day on days 1 and 2; 225 mg/kg on day 3	4 hrs post-dose on days 1 and 2; 24 hrs post-dose on day 3.	Biotransformation map; temporal tracking of parent and biotransformation product levels; endogenous metabolic and lipid signature
KU60648 Rat Cardiac Tissue	Rat – Cardiac Tissue			24 hrs post day 3 dose (i.e., day 4)	Biotransformation map; endogenous metabolic and lipid signature
Sunitinib – cardiomyocytes	Cardiomyocyte intracellular extracts	Sunitinib	5 uM	1, 6 and 24 hrs	Evaluation of metabolite in vitro competency / xenobiotic biotransformation map
	Spent culture media of cardiomyocytes				
Fate of pharmaceuticals in humans	Human - Plasma	Unknown	Unknown	Unknown	Discovering exposure; Biotransformation maps

Supplementary Table 2 Number of features in rat plasma and cardiac tissue untargeted metabolomics datasets before and after application of the three untargeted ADME/TK workflow filters. *Separate full peak matrices per metabolomics assay were produced for sunitinib-exposed and corresponding biological control rat plasma samples, and KU60648-exposed and corresponding biological control rat plasma samples. Also, a single full peak matrix per metabolomics assay comprising data from both sunitinib-exposed and KU60648-exposed, and their corresponding biological control, rat cardiac tissue samples was produced. The number of putative xenobiotic-related features discovered by application of the three intensity-based filters for each xenobiotic are presented, and the number of features retained after removal of those features from the full peak matrices to generate the filtered (endogenous) peak matrices.*

		HILIC positive	HILIC negative	RP C_{18} positive	RP C_{18} negative
**Sunitinib exposed/biological control rat plasma**	Number of features in full peak matrix	12,197	10,920	8,339	6,297
	Number of putative sunitinib-related features	104	7	30	2
	Number of features in filtered (endogenous) peak matrix	12,093	10,913	8,309	6,295
**KU60648 exposed/biological control rat plasma**	Number of features in full peak matrix	12,493	12,318	8,142	5,318
	Number of putative KU60648-related features	147	41	71	17
	Number of features in filtered (endogenous) peak matrix	12,346	12,277	8,071	5,301
**Sunitinib exposed/KU60648 exposed/biological control rat cardiac tissue**	Number of features in full peak matrix	8,202	7,585	11,832	6,273
	Number of putative sunitinib-related features	68	41	137	53
	Number of putative KU60648-related features	71	57	217	63
	Number of features in filtered (endogenous) peak matrix	8,063	7,487	11,478	6,157

Supplementary Table 3 Summary of the two parent pharmaceuticals investigated in rats. *The number of biotransformation products (BTPs) of sunitinib and KU60648 that have been reported in published literature are noted, in addition to how many of those are available to purchase as analytical standards, based on information found within the "Chemical Vendors" section of PubChem records. Also presented are the number of biotransformation products which were predicted by either SyGMa[1] or the "Generate Expected Compounds" tool of Compound Discoverer (Thermo Scientific) and the number of biotransformations discovered in the plasma of exposed rats through application of the untargeted ADME/TK workflow on UHPLC-MS-based metabolomics datasets.*

Parent	Number of BTPs previously reported in literature	Number of BTPs available as analytical standards	Number of *in silico* predicted BTPs	Number of BTPs discovered by untargeted ADME/TK workflow
Sunitinib	11†	4	1492	19
KU60648	0	0	1108	22

† Speed *et al*., 2012[2]

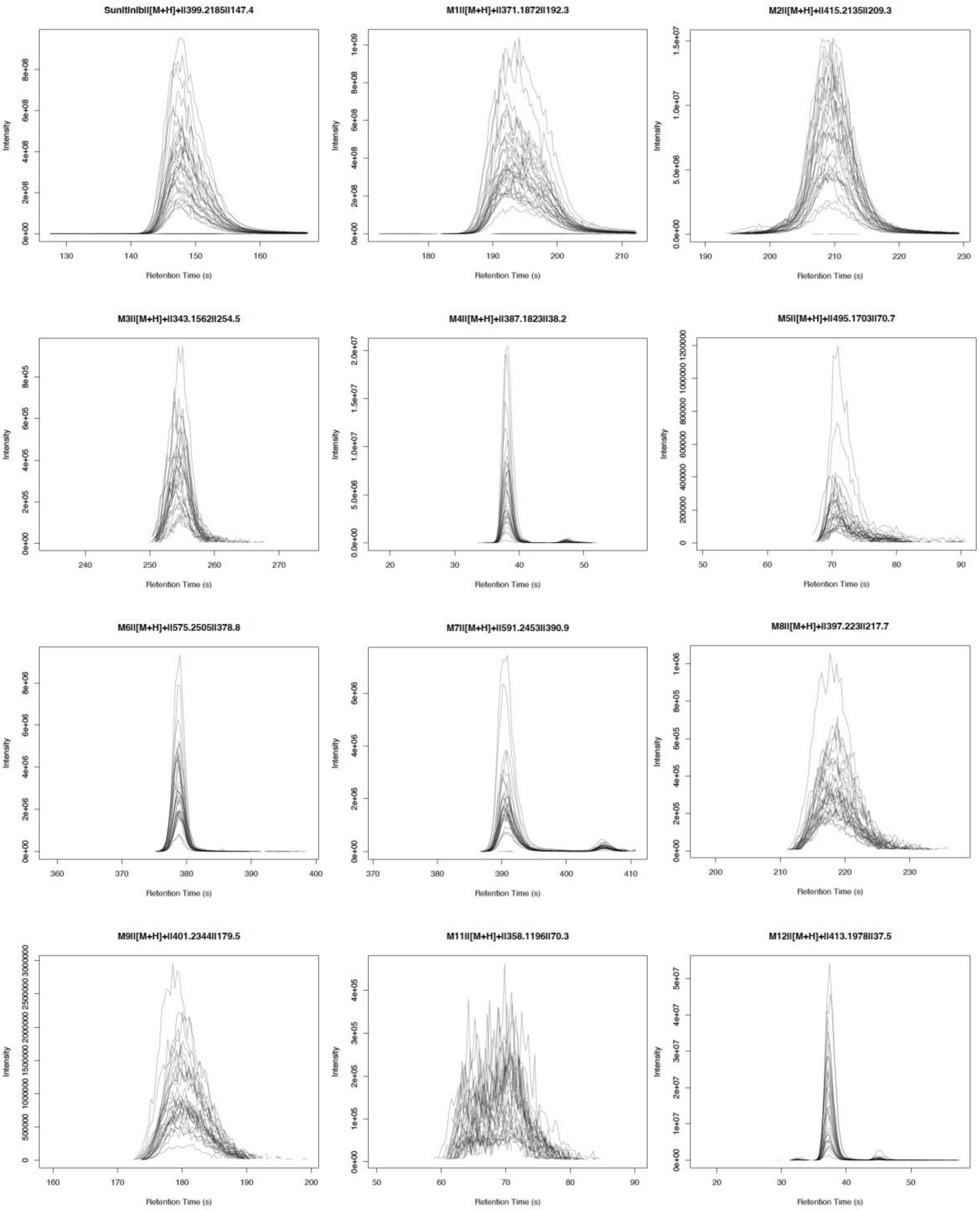
SunItInIbII[M+H]+II399.2185II147.4
M1II[M+H]+II371.1872II192.3
M2II[M+H]+II415.2135II209.3
M3II[M+H]+II343.1562II254.5
M4II[M+H]+II387.1823II38.2
M5II[M+H]+II495.1703II70.7
M6II[M+H]+II575.2505II378.8
M7II[M+H]+II591.2453II390.9
M8II[M+H]+II397.223II217.7
M9II[M+H]+II401.2344II179.5
M11II[M+H]+II358.1196II70.3
M12II[M+H]+II413.1978II37.5
Intensity
Retention Time (s)

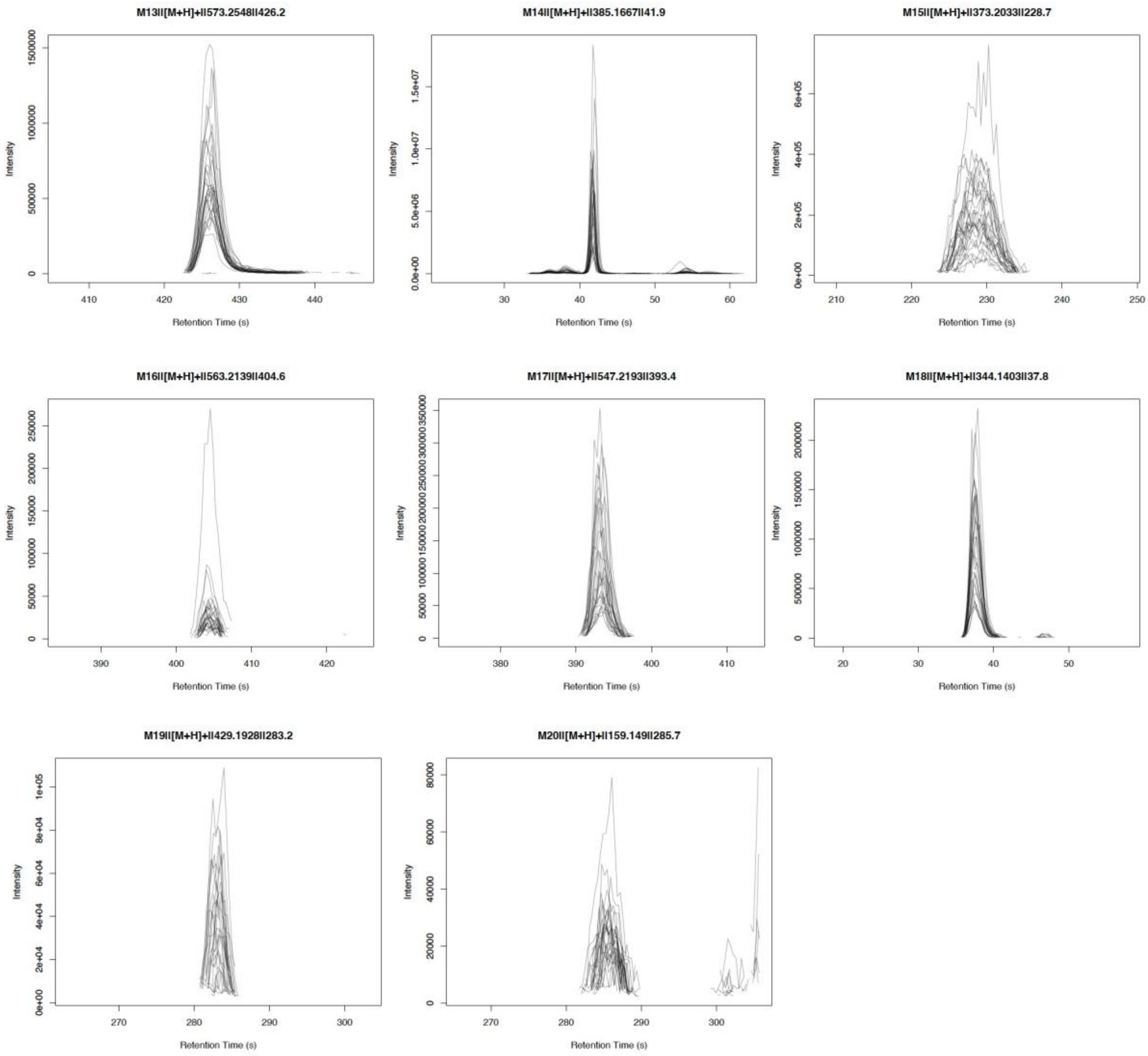

Supplementary Figure 2 Chromatographic peaks of sunitinib and related compounds measured in rat plasma. *Extracted ion chromatograms (EICs) of sunitinib and its biotransformation products measured in the plasma of rats treated with sunitinib by HILIC positive UHPLC-MS untargeted metabolomics.*

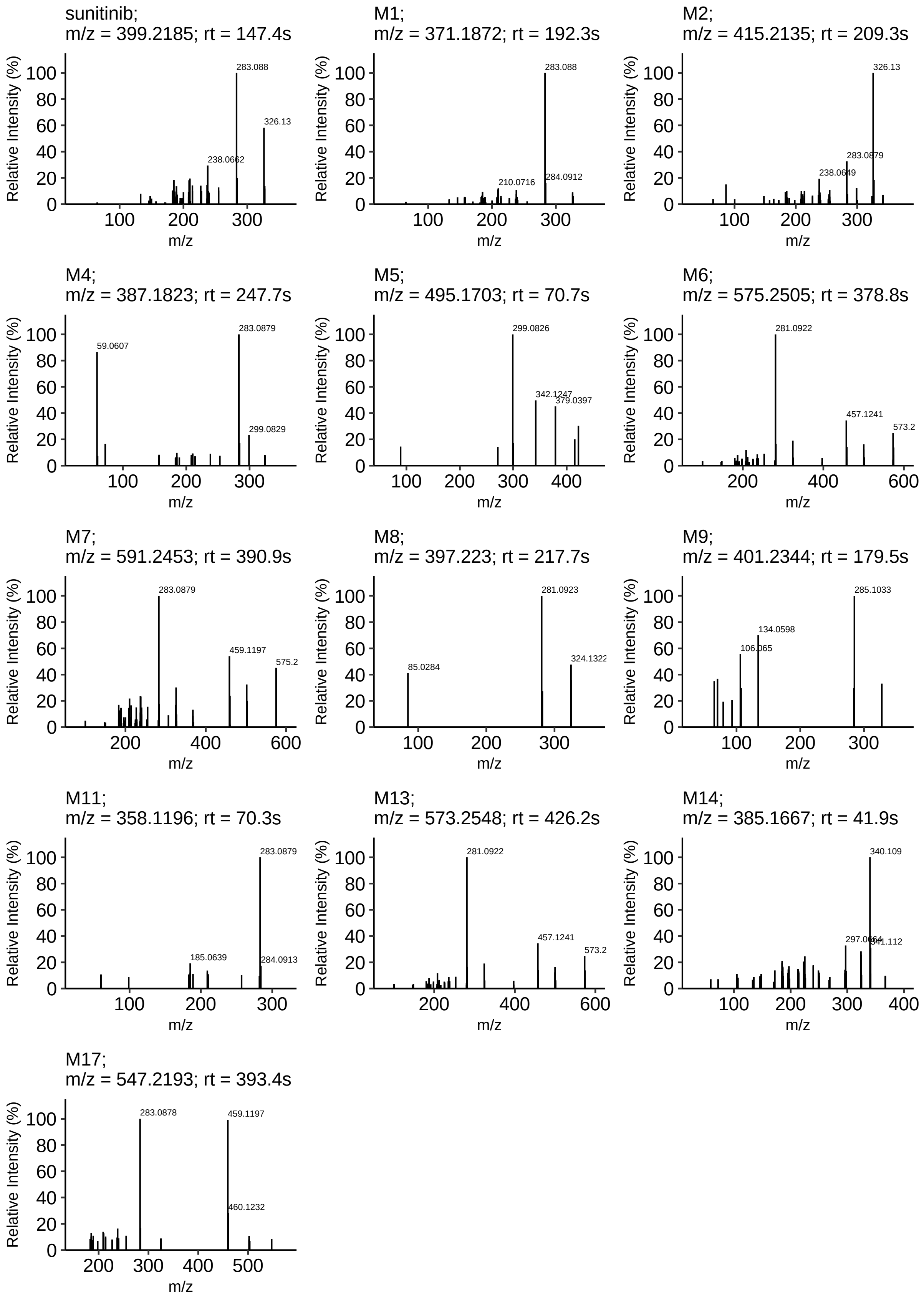

Supplementary Figure 3 Mass spectra of sunitinib biotransformation products measured in rat plasma. *MS^2 fragmentation spectra of biotransformation products of sunitinib measured in the plasma of rats treated with sunitinib by HILIC positive UHPLC-MS untargeted metabolomics.*

Supplementary Note 1 Untargeted workflow discovers fate of KU60648 in rat. *Implementing the untargeted ADME/TK workflow to detect cardiotoxins and their biotransformation products in rat plasma and cardiac tissue – KU60648*

Through application of the workflow, 147, 41, 71 and 17 putative KU60648-related features were extracted from datasets collected by four untargeted metabolomics assays: HILIC-Pos, HILIC-Neg, RP-C_{18}-Pos and RP-C_{18}-Neg, respectively (Supplementary Table 2). Exploratory correlation analysis highlighted the strong association of these features across the exposed rat samples, adding confidence to their predicted relationship to KU60648 (Fig. 3a).

A list of 1108 predicted biotransformation products was generated by *in silico* predictions (Supplementary Data 1) enabling the annotation of 20 biotransformation products from the lists of putative KU60648-related features, as well as KU60648 itself. One further biotransformation product was detected that was not predicted by either SyGMa[1] or the "Generate Expected Compounds" tool of Compound Discoverer (Thermo Scientific) (Fig. 3b). The majority of biotransformation products were detected by the HILIC-Pos assay (Fig. 3c; Supplementary Fig. 4). Furthermore, 17 of the detected biotransformation products were Phase I modifications of KU60648 and 4 were Phase II modifications (Fig. 3d), whilst the biotransformation step(s) that result in the formation of M21 was not elucidated. The identity of features corresponding to KU60648 itself were confirmed by comparison against the MS^2 fragmentation spectra of an authentic chemical standard (Fig. 3e). The MS^2 spectra of KU60648 (from analysis of an authentic chemical standard and exposed rat plasma) were structurally-annotated using MetFrag[3], discovering that peaks at *m/z* 70, 84, 127, 273, 429 and 441 represent major substructures of KU60648. Of the 22 biotransformation products annotated using accurate mass measurements, MS^2 spectra were successfully acquired for 14 and used to confirm the presence of KU60648 substructure(s) and elucidate sites of metabolism by *in silico* structural annotation using MetFrag[3] (Fig. 3f, Supplementary Data 3, Supplementary Fig. 5). This included the 4 compounds (M18, M19, M21, M22) from the list of measured putative KU60648-related features that were not predicted *in silico*. Thus, applying the untargeted ADME/TK workflow, we report the presence of KU60648 to Metabolomics Standards Initiative (MSI)[4] and Schymanski[5] confidence level 1 (using accurate *m/z,* retention time and MS^2 spectral match to authentic standard), and discovery of 14 biotransformation products to MSI level 2 (Schymanski confidence levels 2-3, i.e., probable or tentative structure, using accurate *m/z* and *in silico* structural annotation of MS^2 spectra) and a further 8 biotransformation products with putative molecular formulae (Schymanski confidence level 4, using accurate *m/z*) in the plasma of rats exposed to KU60648 (Fig. 3f, Supplementary Data 3).

The workflow was also applied to untargeted UHPLC-MS metabolomics data collected from cardiac tissue samples taken from the same KU60648-exposed rats, to demonstrate its capability to discover fingerprints of biotransformation at the site of toxicity. Following data filtering (Supplementary Table 2), 4 of the 22 biotransformations detected in plasma were also detected in cardiac tissue (Fig. 3f, Supplementary Data 3). These biotransformations include Phase 1 modifications, amide hydrolysis (M2, M10) and de-ethylation (M14), and a Phase II modification, glucuronide conjugation (M10).

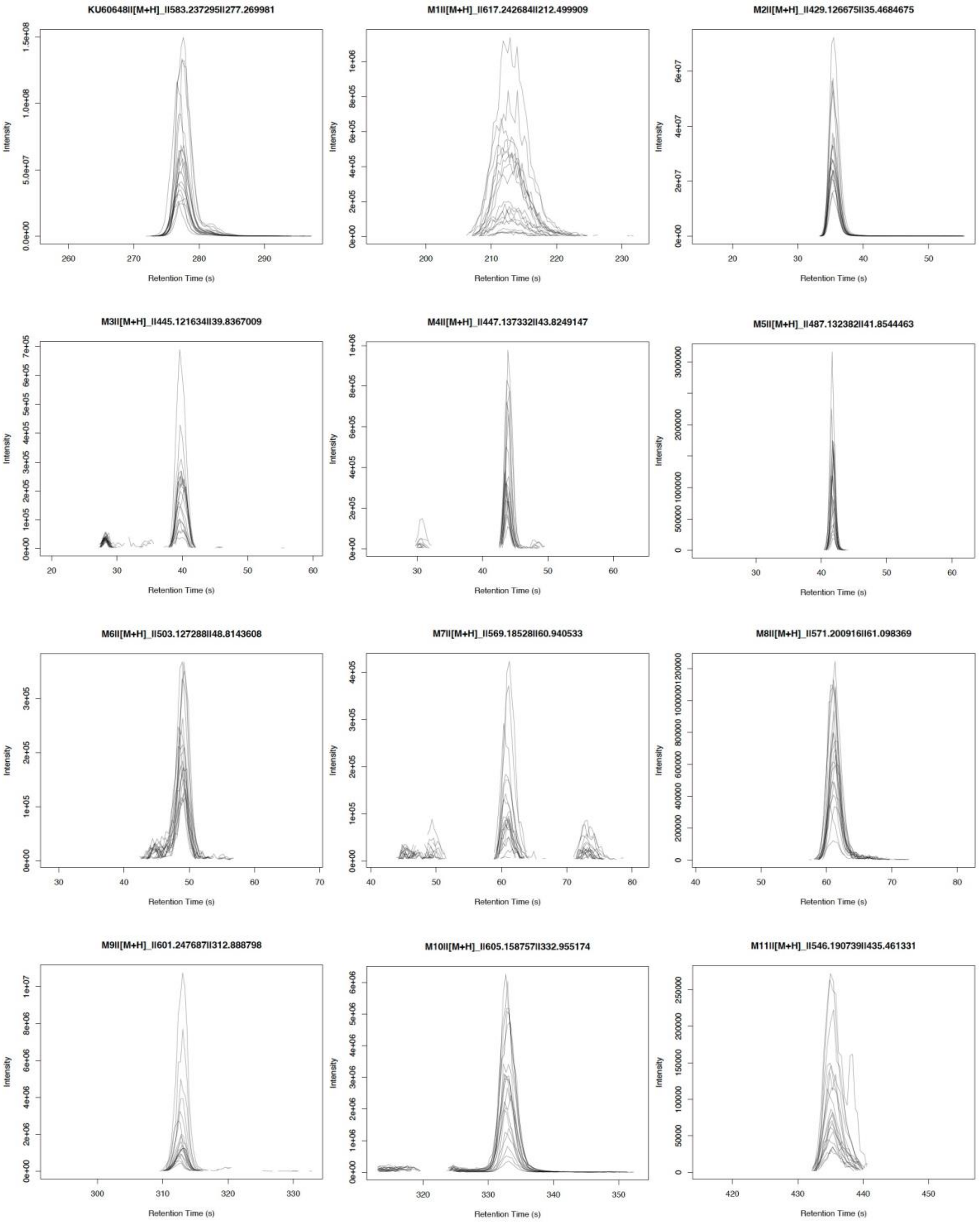
KU60648||[M+H]_||583.237295||277.269981
M1||[M+H]_||617.242684||212.499909
M2||[M+H]_||429.126675||35.4684675
M3||[M+H]_||445.121634||39.8367009
M4||[M+H]_||447.137332||43.8249147
M5||[M+H]_||487.132382||41.8544463
M6||[M+H]_||503.127288||48.8143608
M7||[M+H]_||569.18528||60.940533
M8||[M+H]_||571.200916||61.098369
M9||[M+H]_||601.247687||312.888798
M10||[M+H]_||605.158757||332.955174
M11||[M+H]_||546.190739||435.461331
Intensity
Retention Time (s)

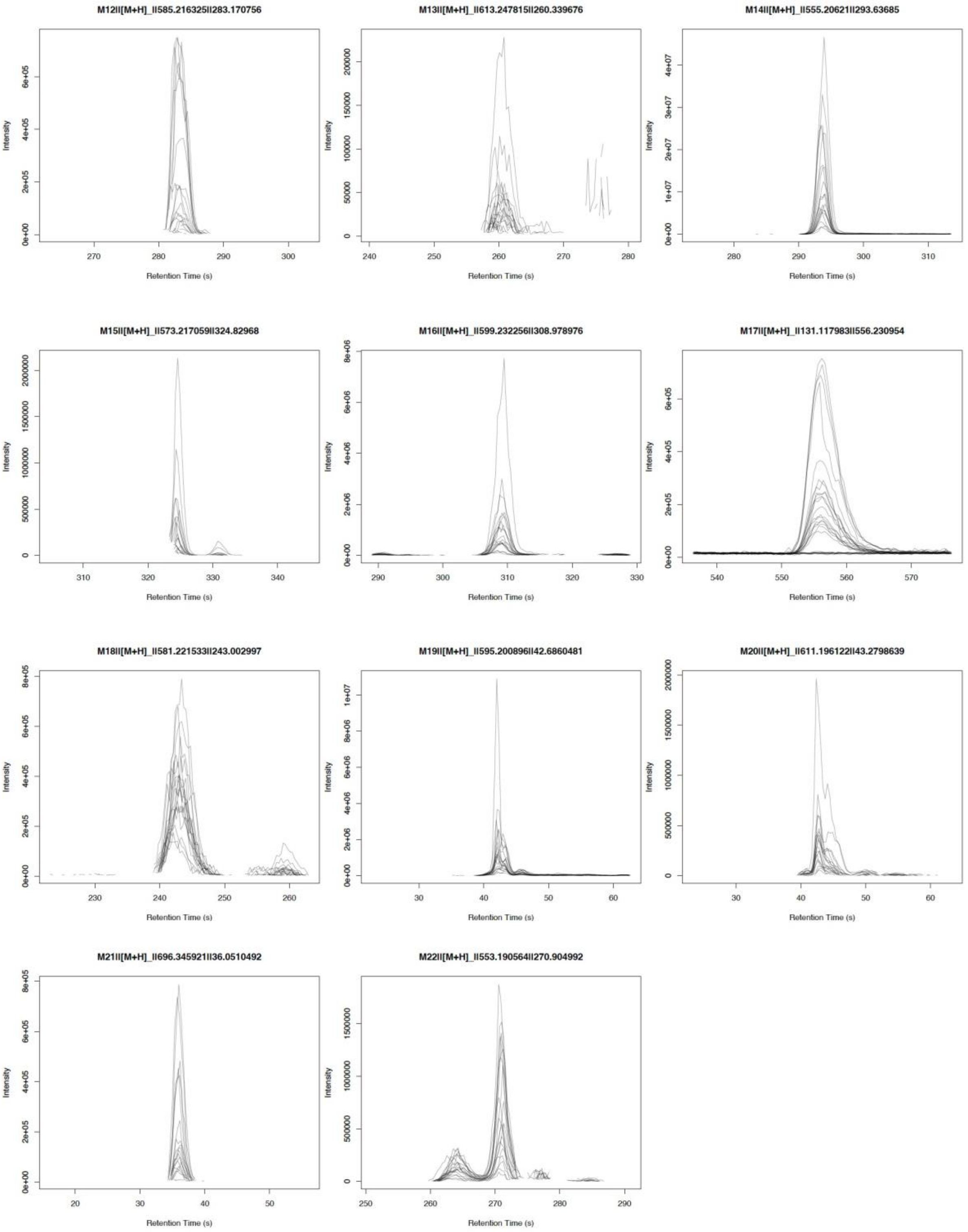

Supplementary Figure 4 Chromatographic peaks of KU6068 and related compounds measured in rat plasma. *Extracted ion chromatograms (EICs) of KU60648 and its biotransformation products measured in the plasma of rats treated with KU60648 by HILIC positive UHPLC-MS untargeted metabolomics.*

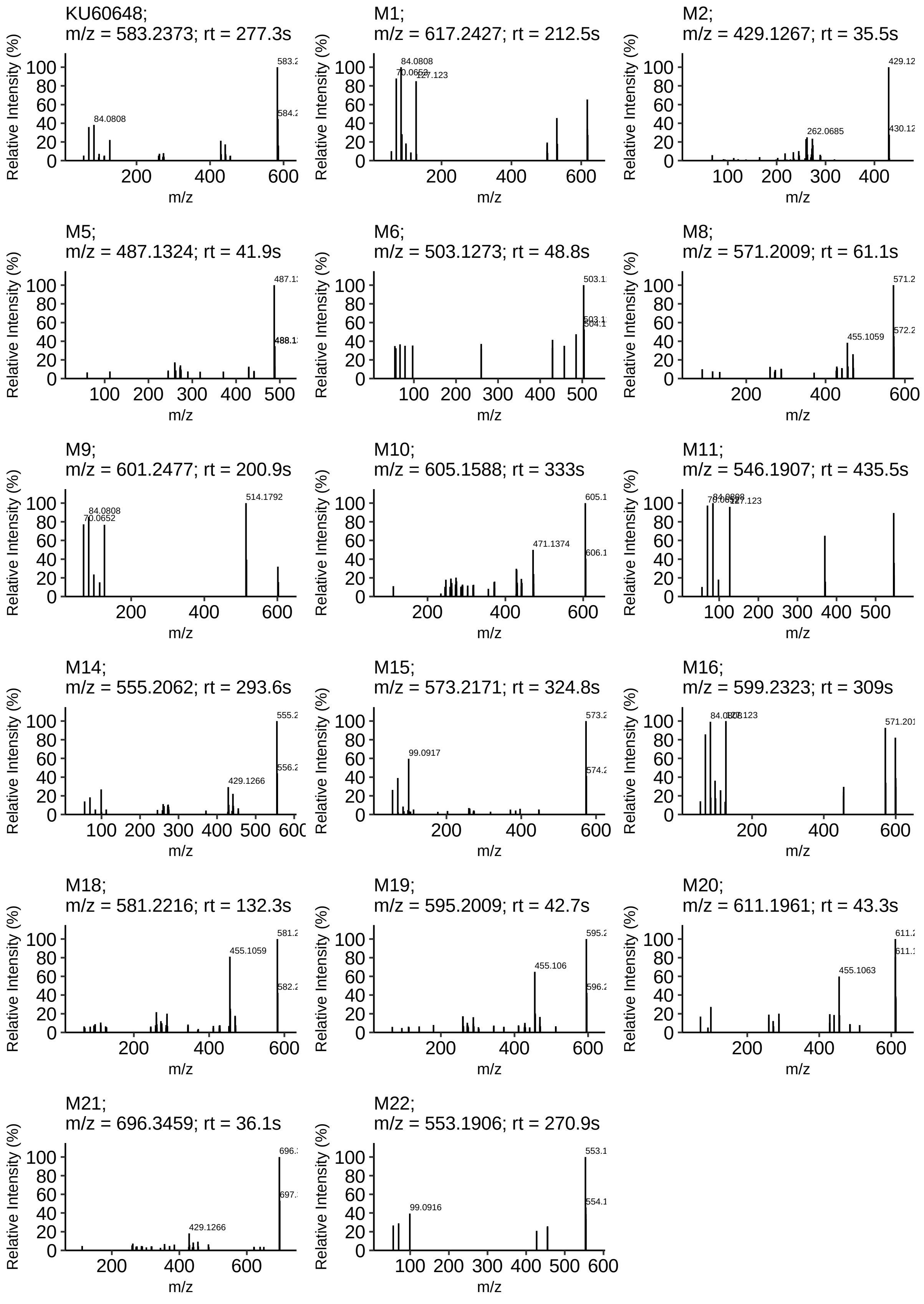

Supplementary Figure 5 Mass spectra of KU60648 biotransformation products measured in rat plasma. *MS^2 fragmentation spectra of the biotransformation products of KU60648 measured in the plasma of rats treated with KU60648 by HILIC positive UHPLC-MS untargeted metabolomics.*

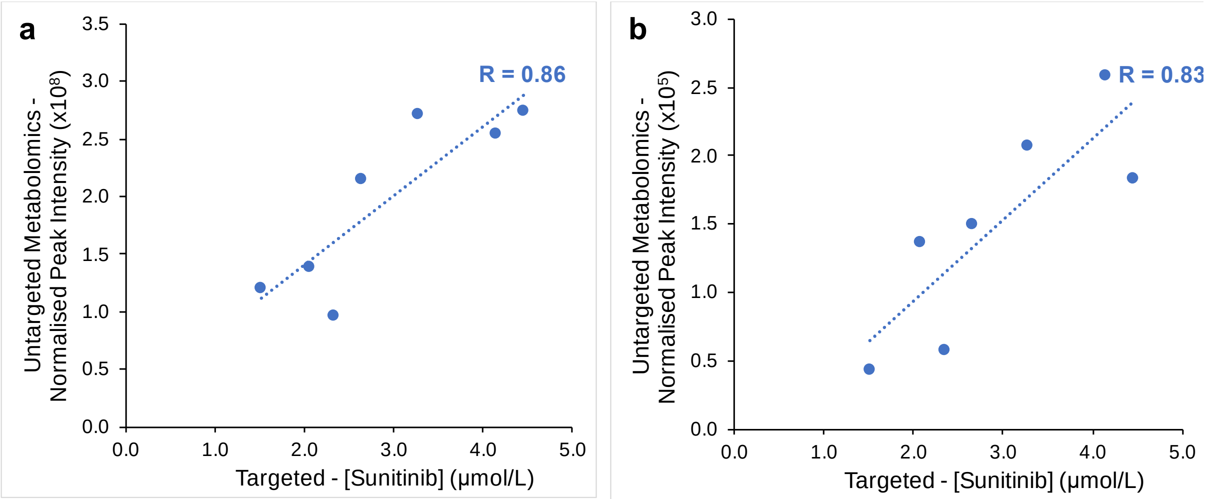

Supplementary Figure 6 Reliability of untargeted metabolomics peak intensity measurements of sunitinib. *Relationship between peak intensity measurements of sunitinib measured by untargeted* ***a*** *RP C_{18} positive and* ***b*** *HILIC negative UHPLC-MS metabolomics in rat plasma sampled on day 15, and quantitative measurements of sunitinib derived from targeted HPLC-MS/MS analysis of rat plasma also sampled on day 15 (4h earlier than metabolomics samples). Pearson correlation coefficient (R) of n = 5 measurements from separate animals is displayed. Source data for this figure are provided in the Source Data file.*

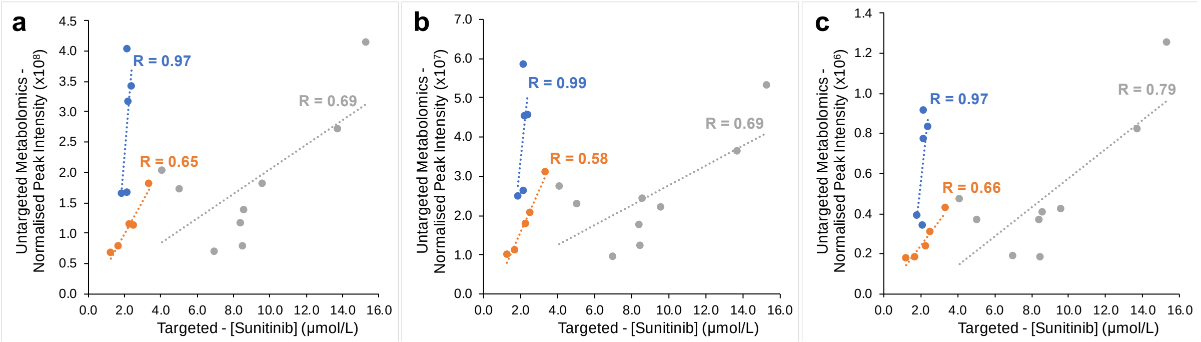

Supplementary Figure 7 Reliability of untargeted metabolomics relative peak intensity measurements of KU60648. *Relationship between peak intensity of KU60648 measured by untargeted* ***a*** *HILIC positive,* ***b*** *RP C_{18} positive, and* ***c*** *HILIC negative UHPLC-MS metabolomics, and quantitative measurements of KU60648 derived from targeted HPLC-MS/MS analysis. Rat plasma for both analyses was sampled at the same time on day 1 (orange). Rat plasma for UHPLC-MS untargeted metabolomics was sampled 20 hrs earlier, and 20 hrs later on days 2 (blue) and 4 (grey), respectively, compared to plasma for targeted HPLC-MS/MS analysis. Pearson correlation coefficients (R) of n = 5 (day 1 and day 2) or n = 8 (day 4) measurements from separate animals are also displayed. Source data for this figure are provided in the Source Data file.*

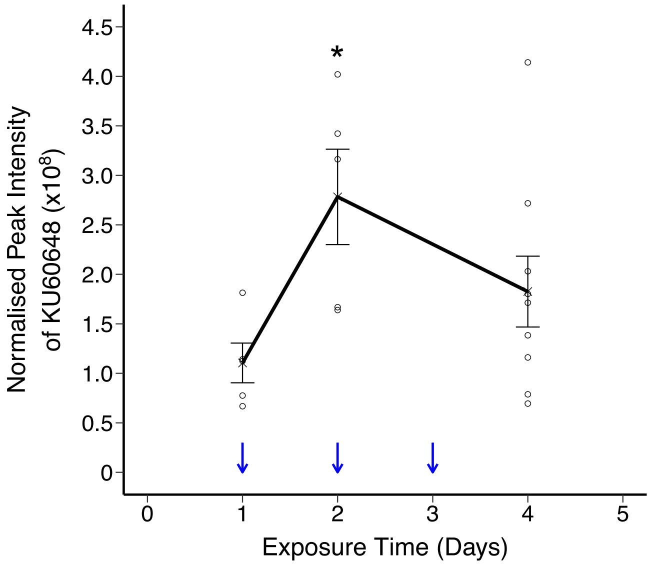

Supplementary Figure 8 Temporal distribution of KU60648 in plasma of exposed rats. *Mean (cross) peak intensity of KU60648, measured by UHPLC-MS untargeted metabolomics, over the duration of the 4-day study. N = 5 separate animals on days 1 and 2; n = 9 separate animals on day 4. Individual data points are also displayed (open circle). Error bars show standard error. Blue arrows indicate time of dosing. Statistical analysis was conducted by one-way ANOVA followed by Tukey's post-hoc test (* p = 0.0344, day 2 vs. day 1). Systemic levels of KU60648 are significantly higher in the plasma of exposed rats by 1.5-fold 4 hrs after a second dose, compared to those observed 4 hrs after the first dose. Meanwhile, on day 4 (24 hrs after the final dose), relative levels lie between those measured on days 1 and 2. The largest inter-individual variation is observed on day 2, when systemic levels of KU60648 are at their highest. Source data for this figure are provided in the Source Data file.*

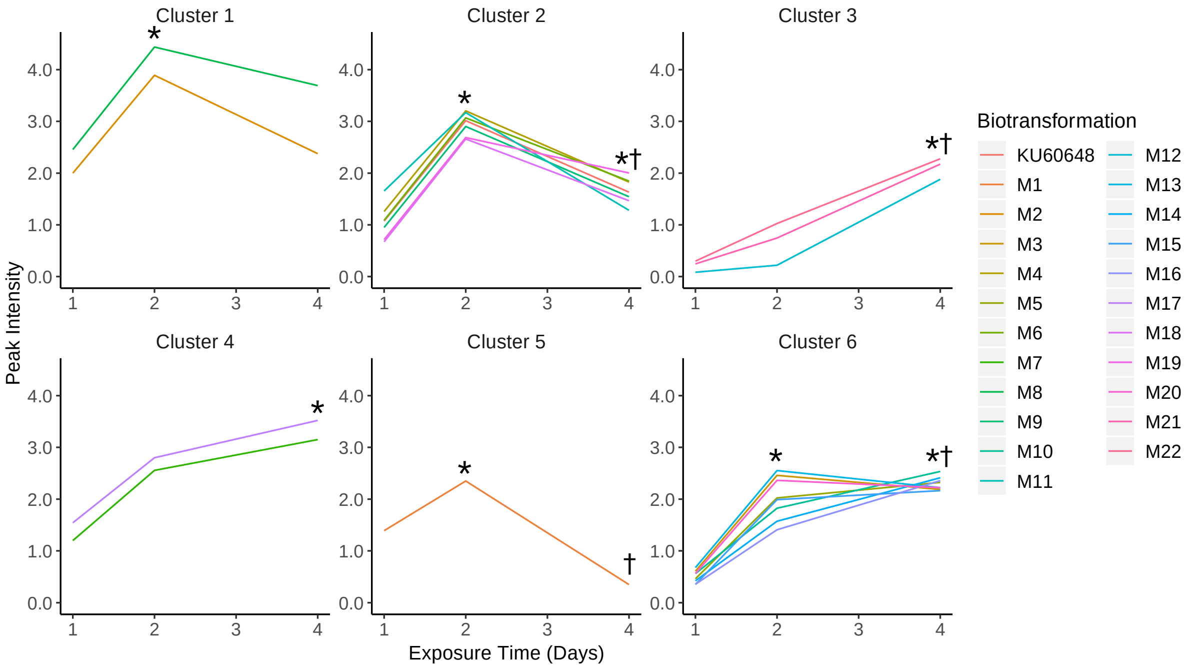

Supplementary Figure 9 Temporal distributions of KU60648 and its biotransformation products in plasma of exposed rats. *Median peak intensities, measured by UHPLC-MS untargeted metabolomics and scaled by unit-variance, of KU60648 and its biotransformation products over the duration of the 4-day study, clustered by unsupervised k-means clustering (k = 6, optimal value determined by the Elbow Method). Statistical analysis was conducted by one-way ANOVA followed by Tukey's post-hoc test, with significance displayed as follows: * p < 0.05 vs. day 1, † p < 0.05 vs. day 2, ‡ p < 0.05 vs. day 4. Specifically, cluster 1: p = 0.0002 (day 2 vs. day 1); cluster 2: p < 0.0001 (day 2 vs. day 1), p = 0.0005 (day 4 vs, day 1), p = 0.0132 (day 4 vs. day 2); cluster 3: p < 0.0001 (day 4 vs. day 1 and day 4 vs. day 2); cluster 4: p = 0.0013 (day 4 vs. day 1); cluster 5: p = 0.0307 (day 2 vs. day 1), p = 0.0003 (day 4 vs. day 2); cluster 6: p = 0.0001 (day 2 vs. day 1), p < 0.0001 (day 4 vs. day 1), p = 0.0148 (day 4 vs. day 2). N = 5 separate animals on days 1 and 2; n = 9 separate animals on day 4. KU60648 is clustered with 6 of its biotransformation products: M4, M6, M9, M11, M18 and M19 (cluster 2). The average response of these compounds shows a significant and large increase (2.8-fold) in systemic levels between days 1 and 2 followed by a slight, but significant, decline by day 4, although levels remain significantly 1.5-fold greater than those measured on day 1. Cluster 1 (M2 and M8) shows a similar temporal trend to Cluster 2, however the increase between day 1 and 2 is to a lesser extent (1.8-fold compared to 2.8-fold). Cluster 3 (M12, M21, and M22) shows consistent accumulation throughout the experiment. The accumulation is significant by day 4 where levels were 9-fold greater than those measured on day 1. Cluster 4 (M7 and M17) has a similar response to Cluster 3, with significant change occurring by day 4. However, this accumulation is lesser, with day 4 levels 2.5-fold greater than those measured on day 1. M1 does not cluster with any other KU60648 biotransformation products (cluster 5). Its systemic levels significantly increase between days 1 and 2 to levels 1.7-fold greater but returns to levels comparable to day 1 by day 4. Cluster 6 (M3, M5, M10, M13, M14, M15, M16 and M20) systemic levels are significantly 4-fold greater on day 2 than on day 1, with a further 2-fold increase by day 4. Source data for this figure are provided in the Source Data file.*

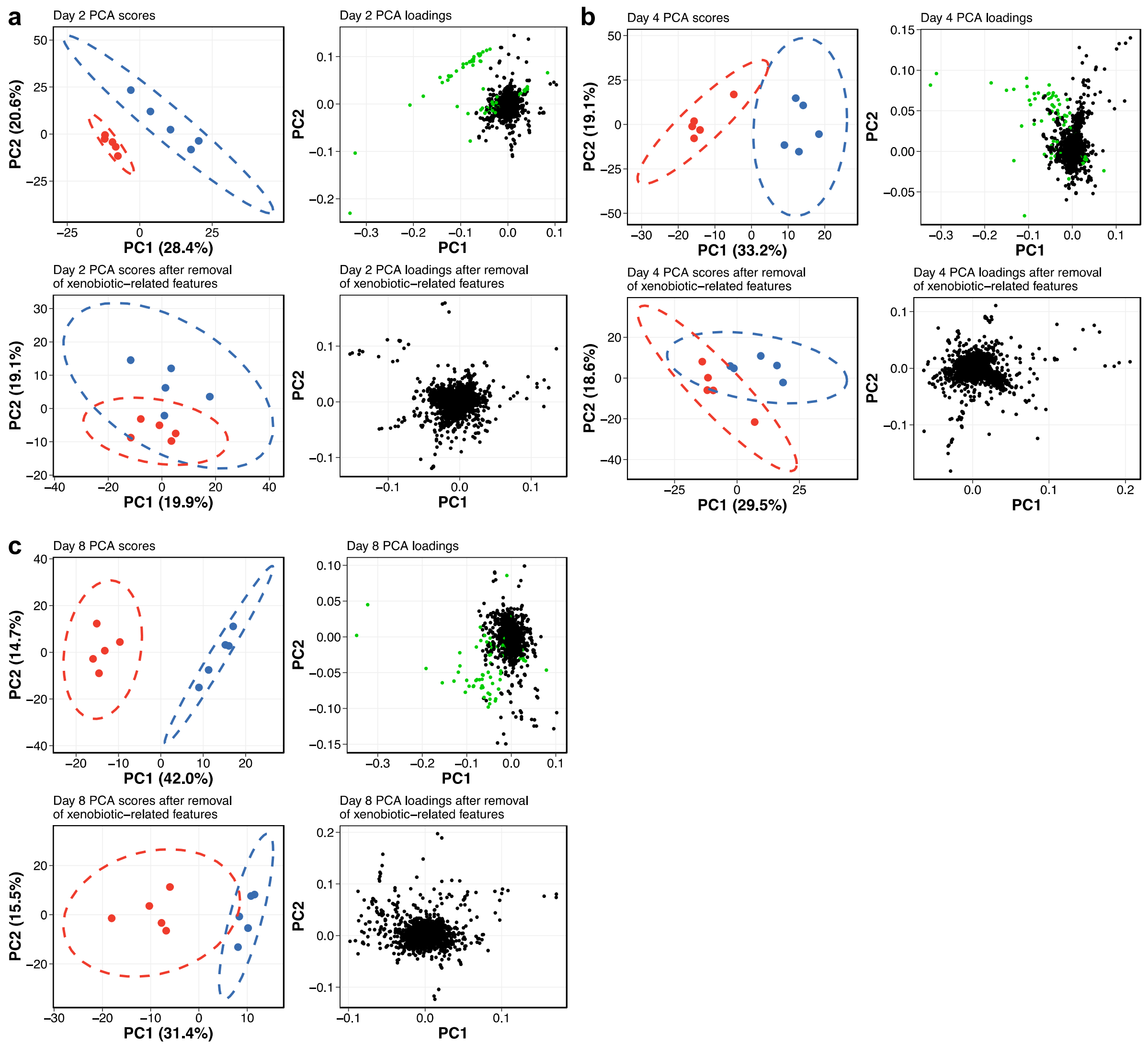

Supplementary Figure 10 Sunitinib-induced perturbation of the plasma metabolome of rats. *PCA scores and loadings plots of data acquired from plasma of rats exposed to sunitinib (red) and biological control rats (blue), after* ***a*** *2 days,* ***b*** *4 days and* ***c*** *8 days, measured by HILIC UHPLC-MS in positive ion mode before and after removal of putative sunitinib-related features (green in the loadings plots).*

Supplementary Table 4 Number of significantly changing features in plasma of rats exposed to sunitinib compared to biological controls, per time point and per analytical assay. *Differences between exposed and biological control samples were evaluated using two-tailed Student's t-test with FDR correction (N = 5 separate animals for days 1, 2, 4 and 8, N = 9 separate animals for day 15). A significant difference was defined where q-value < 0.1.*

Assay	Number of significant features				
	Day 1	Day 2	Day 4	Day 8	Day 15
HILIC positive	25	38	9	109	524
HILIC negative	3	81	1	52	518
RP C_{18} positive	12	2	38	75	1389
RP C_{18} negative	0	0	83	227	1063

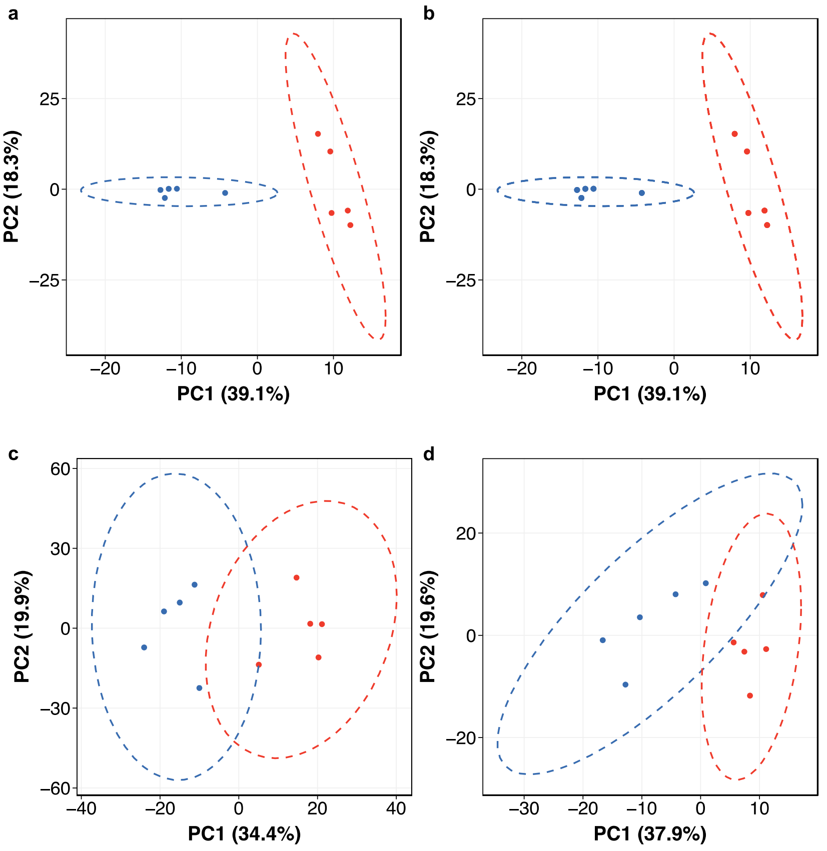

Supplementary Figure 11 Sunitinib-induced perturbation of the cardiac tissue metabolome of rats. *PCA scores plots of cardiac tissue samples from rats exposed to sunitinib (red) compared to biological control rats (blue), as measured by 4 untargeted metabolomics assays:* ***a*** *HILIC UHPLC-MS in positive ion mode,* ***b*** *HILIC UHPLC-MS in negative ion mode,* ***c*** *RP C_{18} UHPLC-MS in positive ion mode and* ***d*** *RP C_{18} UHPLC-MS in negative ion mode. Analysis was carried out on the endogenous data matrices following removal of putative sunitinib-related features.*

Supplementary Table 5 Number of significantly changing features in cardiac tissue of rats exposed to sunitinib compared to biological controls, per assay. *Differences between exposed and biological control samples were evaluated using two-tailed Student's t-test with FDR correction (N = 5 separate animals). A significant difference was defined where q-value < 0.1.*

Assay	Number of significant features
HILIC positive	8
HILIC negative	69
RP C_{18} positive	222
RP C_{18} negative	156

Supplementary Table 6 Over-representation analysis of lipids significantly correlated to sunitinib levels in the cardiac tissue of exposed rats. *The number of lipid species per lipid class annotated to MSI level 2 (Reference List Count), and number of those lipids significantly correlated (Spearman's, p < 0.05) to sunitinib levels in exposed samples (network count) are reported. Expected network count refers to the number of species per lipid class expected to be significantly correlated to sunitinib levels in exposed samples in the case of no lipid class enrichment. The fold enrichment of lipid classes significantly correlated (Spearman's, p < 0.05) to sunitinib levels in exposed samples was calculated as the ratio of network count to expected network count, and the significance assessed by a one-sided Fisher's Exact test (p-value).*

Lipid Class	Reference List Count	Network Count	Expected Network Count	Fold Enrichment	p-value
**Cer**	28	7	3.94	1.78	0.064
**LPE**	3	2	0.42	4.76	0.053
**MePC**	6	1	0.84	1.19	0.595
**PC**	107	10	15.1	0.66	0.828
**PE**	101	15	14.2	1.06	0.174
**PS**	7	2	0.99	2.02	0.252
**SM**	43	11	6.05	1.82	0.014
**SPH**	1	1	0.14	7.14	0.140
**TG**	142	19	20.0	0.95	0.174

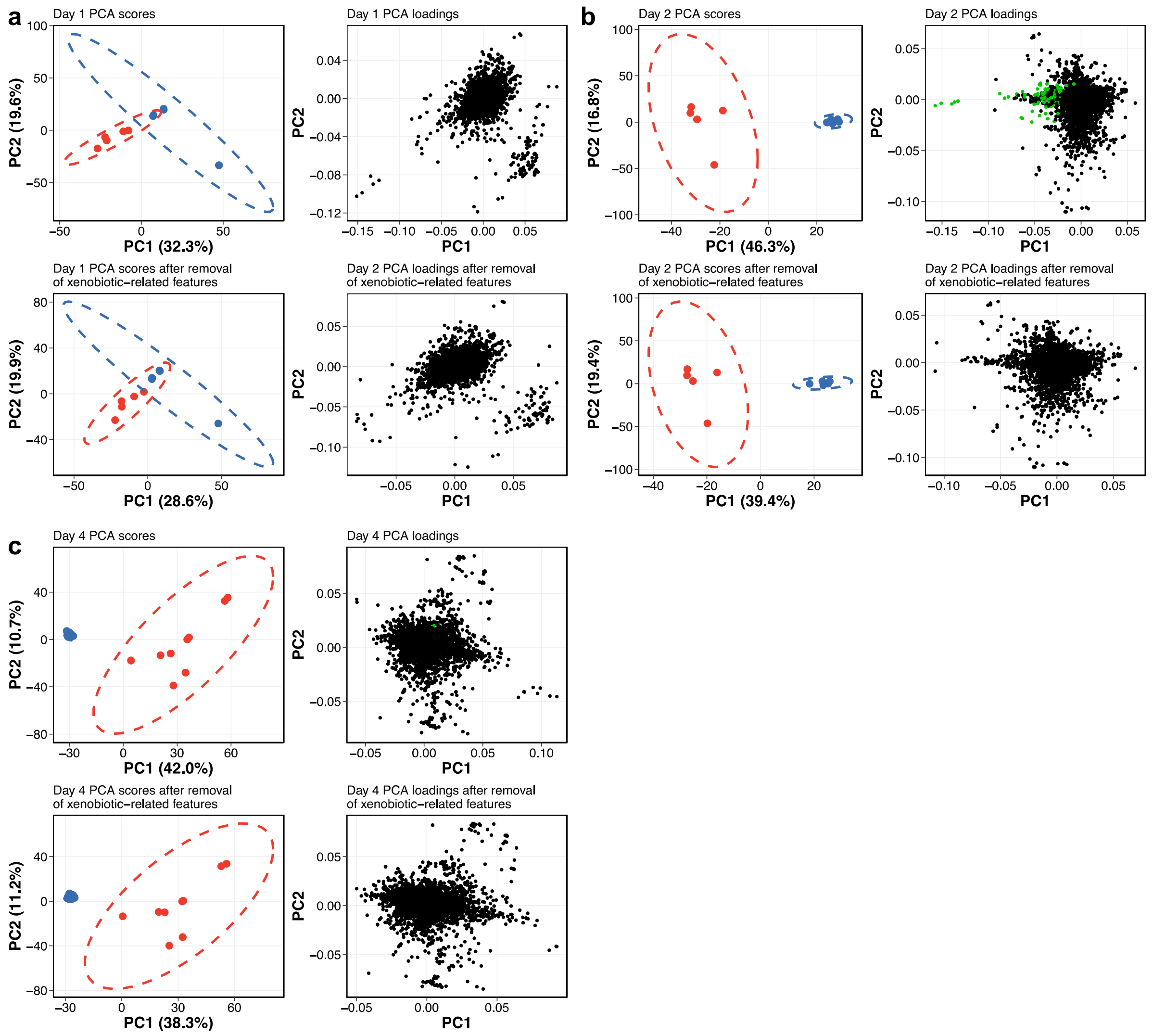

Supplementary Figure 12 KU60648-induced perturbation of the plasma metabolome of rats. *PCA scores and loadings plots of data acquired from plasma of rats exposed to KU60648 (red) and biological control rats (blue), after* ***a*** *1 day,* ***b*** *2 days and* ***c*** *4 days, measured by HILIC UHPLC-MS in positive ion mode before and after removal of putative sunitinib-related features (green in the loadings plots).*

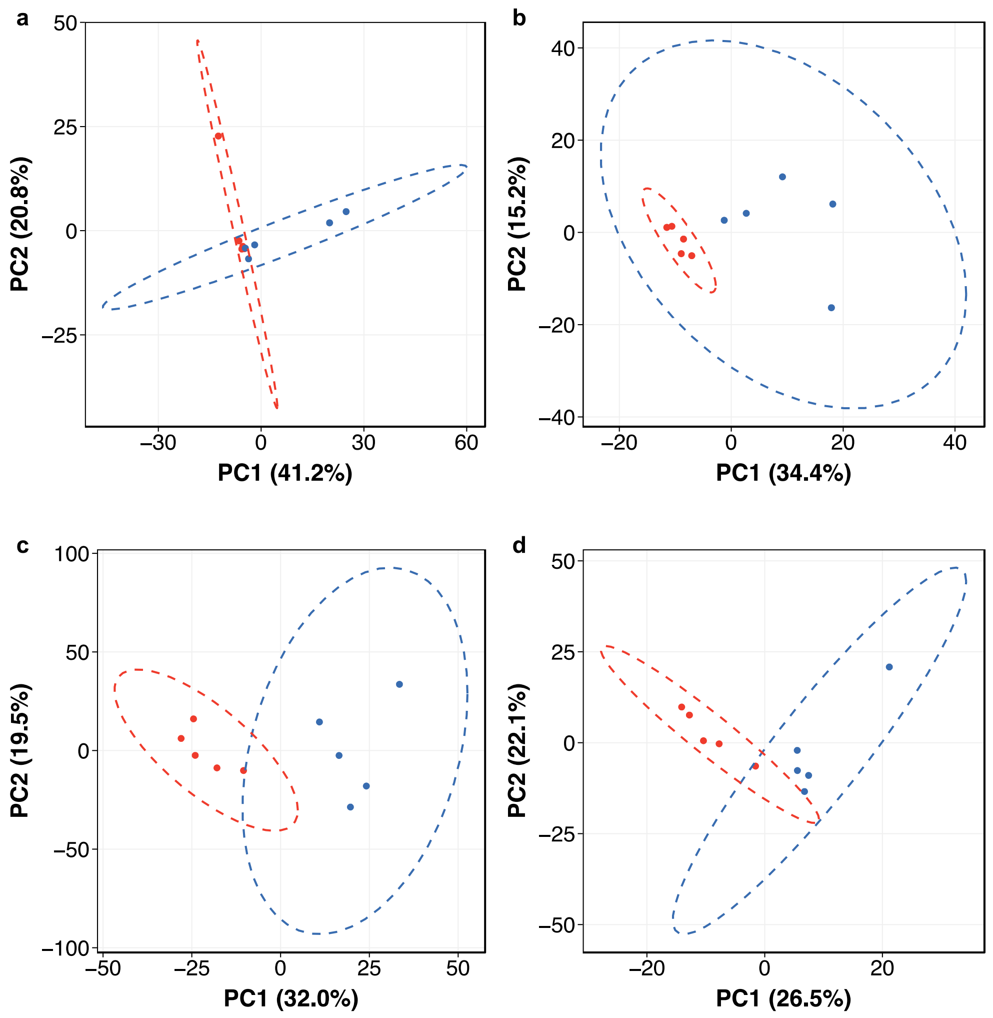

Supplementary Figure 13 KU60648-induced perturbation of the cardiac tissue metabolome of rats. *PCA scores plots of cardiac tissue samples from rats exposed to KU60648 (red) compared to biological control rats (blue), as measured by 4 untargeted metabolomics assays:* ***a*** *HILIC UHPLC-MS in positive ion mode,* ***b*** *HILIC UHPLC-MS in negative ion mode,* ***c*** *RP C_{18} UHPLC-MS in positive ion mode and* ***d*** *RP C_{18} UHPLC-MS in negative ion mode. Analysis was carried out on the endogenous data matrices following removal of putative KU60648-related features.*

Supplementary Table 7 Number of significantly changing features in plasma, per time point, and cardiac tissue of rats exposed to KU60648 compared to biological controls, per time point and per analytical assay*. Differences between exposed and biological control samples were evaluated using two-tailed Student's t-test with FDR correction (plasma: N = 5 separate animals for days 1, and 2, N = 9 separate animals for day 4; cardiac tissue: N = 5 separate animals). A significant difference was defined where q-value < 0.1.*

Assay	Number of significant features			
	Day 1	Day 2	Day 4	Cardiac Tissue
HILIC positive	137	1429	2420	26
HILIC negative	91	1000	2368	81
RP C18 positive	161	2244	2920	944
RP C18 negative	1	1098	2014	496

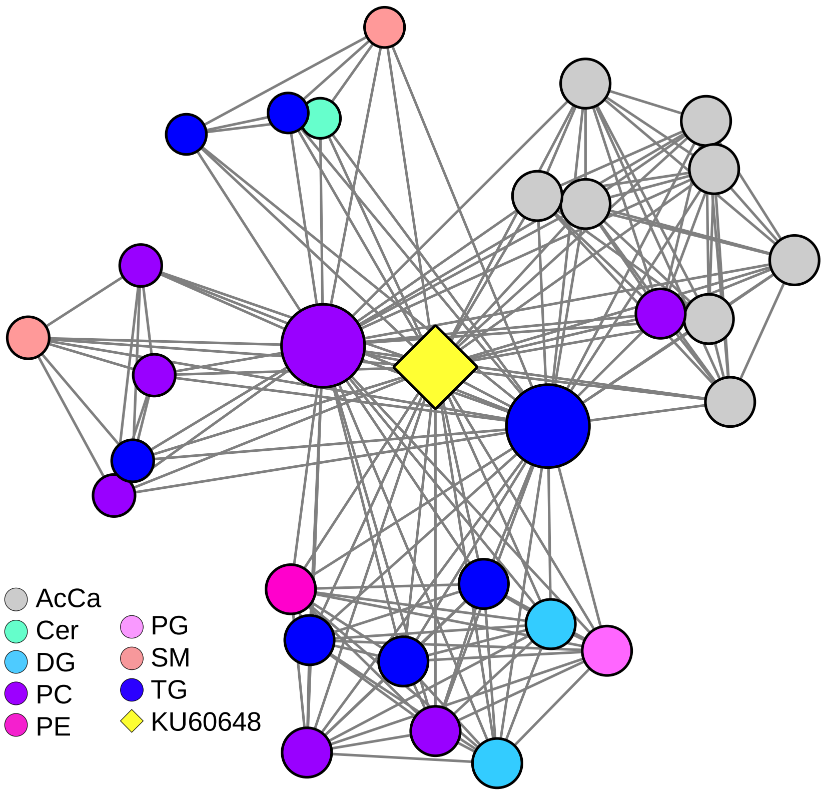

Supplementary Figure 14 Association of internal relative dose of KU60648 with the cardiac tissue lipidome of exposed rats. *Correlation network of KU60648 and MSI level 2 annotated lipids: acylcarnitines (AcCa), ceramides (Cer), diacylglycerols (DG), phosphatidylcholines (PC), phosphatidylethanolamines (PE), phosphatidylglycerol (PG), sphingomyelins (SM) and triacylglycerols (TG), in cardiac tissue of rats exposed to KU60648 (N = 5 individual animals). Node size is proportional to its degree of connectivity. Edges represent significant Spearman's correlation (p-value < 0.05 and, |ρ| ≥ 0.9) between compounds. All nodes displayed are significantly correlated to KU60648.*

Supplementary Table 8 Over-representation analysis of lipids significantly correlated to KU60648 levels in the cardiac tissue of exposed rats. *The number of lipid species per lipid class annotated to MSI level 2 (Reference List Count), and number of those lipids significantly correlated (Spearman's, p<0.05) to KU60648 levels in exposed samples (network count) are reported. Expected network count refers to the number of species per lipid class expected to be significantly correlated to KU60648 levels in exposed samples in the case of no lipid class enrichment. The fold enrichment of lipid classes significantly correlated (Spearman's, p<0.05) to KU60648 levels in exposed samples was calculated as the ratio of network count to expected network count, and the significance assessed by a one-sided Fisher's Exact test (p-value).*

Lipid Class	Reference List Count	Network Count	Expected Network Count	Fold Enrichment (Actual/ Expected)	p-value
**AcCa**	15	8	0.8	10.0	1.467×10^{-7}
**Cer**	28	1	1.6	0.63	0.794
**DG**	13	2	0.7	2.86	0.156
**PC**	107	7	6.0	1.17	0.217
**PE**	101	1	5.7	0.18	0.995
**PG**	6	1	0.3	3.33	0.292
**SM**	42	2	2.4	0.83	0.661
**TG**	142	7	8.0	0.88	0.442

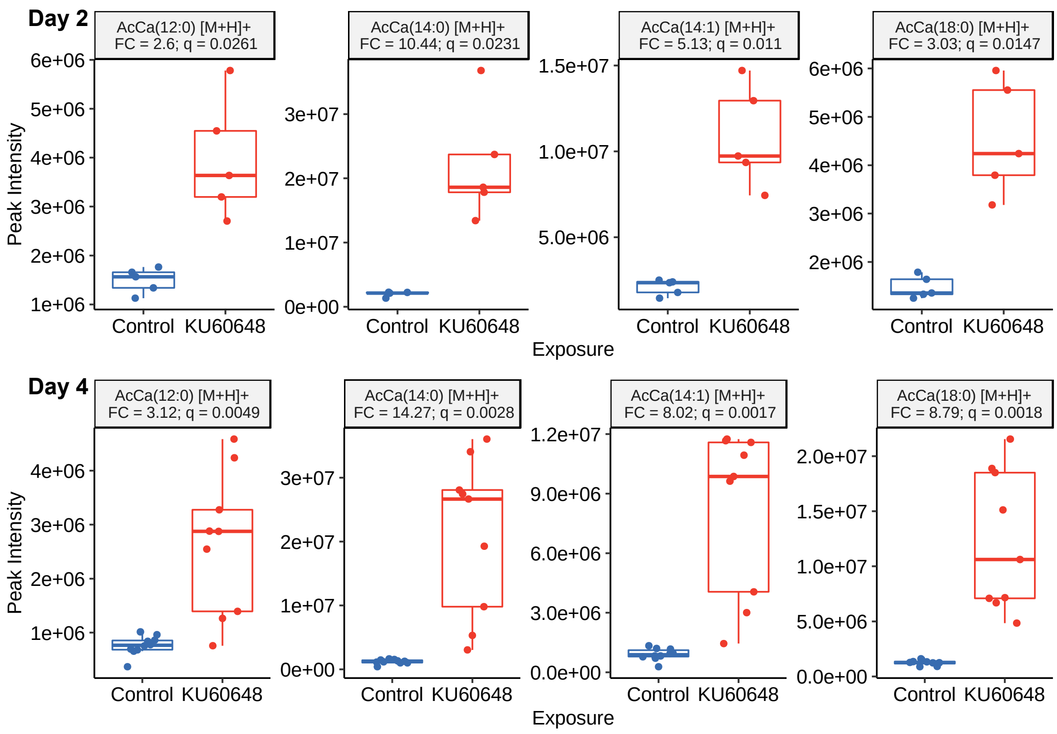

Supplementary Figure 15 Boxplots showing significantly increased plasma levels of 4 acylcarnitines found to be associated with KU60648 levels at the site of toxicity in rats, after 2 and 4 days of exposure to KU60648. *Boxplots showing the peak intensity values of 4 acylcarnitines; AcCa(12:0), AcCa(14:0), AcCa(14:1) and AcCa(18:0), in the plasma of rats exposed to KU60648 for either 2 or 4 days, compared to time-matched biological controls. The levels of the same four acylcarnitines were found to be significantly correlated with levels of KU60648 in the cardiac tissue of rats exposed to KU60648 for 4 days. Boxes show the interquartile range (IQR), with the line representing the median, and the whiskers showing 1.5× IQR. Day 2 and 4 data is from n = 5 and n = 9 individual animals, respectively. Fold changes and q-values (FDR-corrected p-values from Student's two-tailed t-test) are also displayed. Source data for this figure are provided in the Source Data file.*

Supplementary Table 9 Number of features in the untargeted metabolomics datasets of intracellular extracts and culture medium of hiPSC-CM cultures before and after application of the three untargeted ADME/TK workflow filters.

		HILIC positive	HILIC negative	RP C_{18} Positive	RP C_{18} Negative
**Intracellular extracts**	Number of features in full peak matrix	12,028	5,924	8,025	3,690
	Number of putative sunitinib-related features	217	12	102	25
**Culture medium**	Number of features in full peak matrix	6,027	3,426	3,168	1,195
	Number of putative sunitinib-related features	71	3	13	0

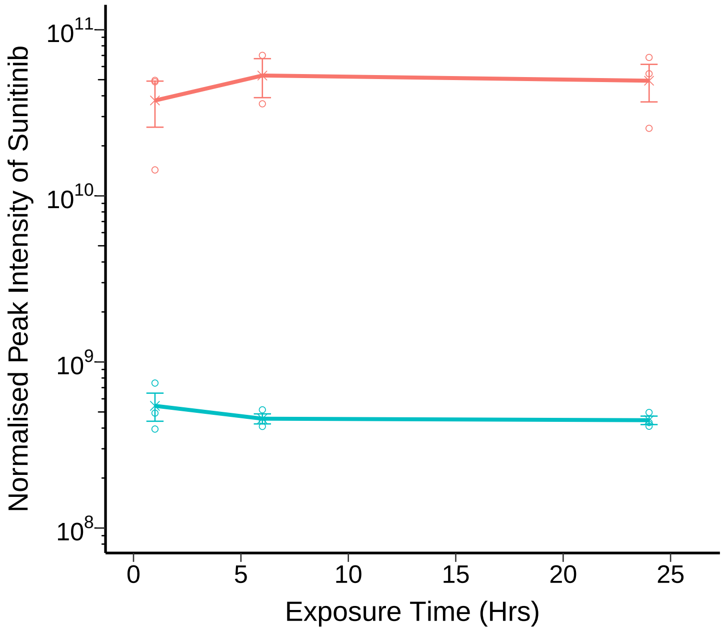

Supplementary Figure 16 Temporal distribution of sunitinib in the intracellular extracts (coral) and culture medium (light blue) of hiPSC-CM cultures exposed to sunitinib. *Mean (cross) peak intensity of sunitinib, measured by UHPLC-MS untargeted metabolomics, over the duration of the 24hr study. N = 3 from separate vials of hiPSC-CMs. Individual data points are also displayed (open circle). Error bars show standard error. Neither intracellular nor extracellular levels of sunitinib significantly change over the duration of the 24hr exposure (one-way ANOVA, p>0.05). Source data for this figure are provided in the Source Data file* .

Supplementary Table 10 UK NHS Top20 most prescribed pharmaceuticals 2014 - 2020 and their detection. *The top 20 most prescribed pharmaceuticals, according to NHS reports from 2014, 2017 and 2020 are presented, alongside their molecular formula and NORMAN database identifier. Details of their measurement in human plasma samples by UHPLC-MS untargeted metabolomics, including the m/z, retention time (RT), ion form, and confidence of annotation (MSI annotation level) are also reported.*

Overall Rank	Pharmaceutical	2014 Rank	2017 Rank	2020 Rank	Molecular Formula	NORMAN	CECscreen	m/z	RT (secs)	Ion Form	MSI Annotation Level	Schymanski Annotation Level
1	Paracetamol	1	1	1	C8H9NO2	NS00000231	Y	152.0707	31.8	$[M+H]^+$	1	1
2	Metformin hydrochloride	3	2	2	C4H11N5	NS00000333	Y	300.1595	24.5	$[M+H]^+$	MS^1 only	4
3	Codeine phosphate	4	3	3	C18H21NO3	NS00000391	Y	Not detected				
4	Lactulose	2	4	6	C12H22O11	Not in database	Y	365.1056	446.5	$[M+Na]^+$	MS^1 only	4
5	Methadone hydrochloride	5	6	5	C21H27NO	NS00000411	Y	332.1988	353.9	$[M+Na]^+$	MS^1 only	4
6	Omeprazole	8	8	7	C17H19N3O3S	NS00003072	Y	346.1221	33.9	$[M+H]^+$	2	2a
7	Atorvastatin	15	7	4	C33H35FN2O5	NS00009054	Y	559.2601	34.0	$[M+H]^+$	2	2a
8	Levothyroxine sodium	7	9	8	C15H11I4NO4	Not in database	Y	Not detected				
9	Cholecalciferol	9	10	9	C27H44O	NS00077941	Y	385.3468	25.4	$[M+H]^+$	MS^1 only	4
10	Ramipril	11	11	11	C23H32N2O5	NS00004700	Y	417.2386	39.8	$[M+H]^+$	1	1
11	Amlodipine	12	12	10	C20H25ClN2O5	NS00000043	Y	Not detected				
12	Simvastatin	6	13	18	C25H38O5	NS00008782	Y	419.2794	27.6	$[M+H]^+$	MS^1 only	4
13	Lansoprazole	16	14	12	C16H14F3N3O2S	NS00000543	Y	370.0834	30.5	$[M+H]^+$	1	1
14	Aspirin	10	16	17	C9H8O4	NS00015094	Y	Not detected				
15	Morphine sulfate	18	18	14	C17H19NO3	NS00093493	Y	286.1439	27.0	$[M+H]^+$	2	2a
16	Bisoprolol fumarate	21	15	13	C18H31NO4	NS00009929	Y	326.2327	95.0	$[M+H]^+$	2	2a
17	Gabapentin	24	17	15	C9H17NO2	NS00000149	Y	172.1334	844.0	$[M+H]^+$	MS^1 only	4
18	Tramadol hydrochloride	13	19	21	C16H25NO2	NS00000338	Y	Not detected				
19	Amitriptyline hydrochloride	20	20	16	C20H23N	NS00000032	Y	278.1904	67.0	$[M+H]^+$	1	1
20	Ibuprofen	17	21	22	C13H18O2	NS00035725	Y	207.1380	27.5	$[M+H]^+$	MS^1 only	4

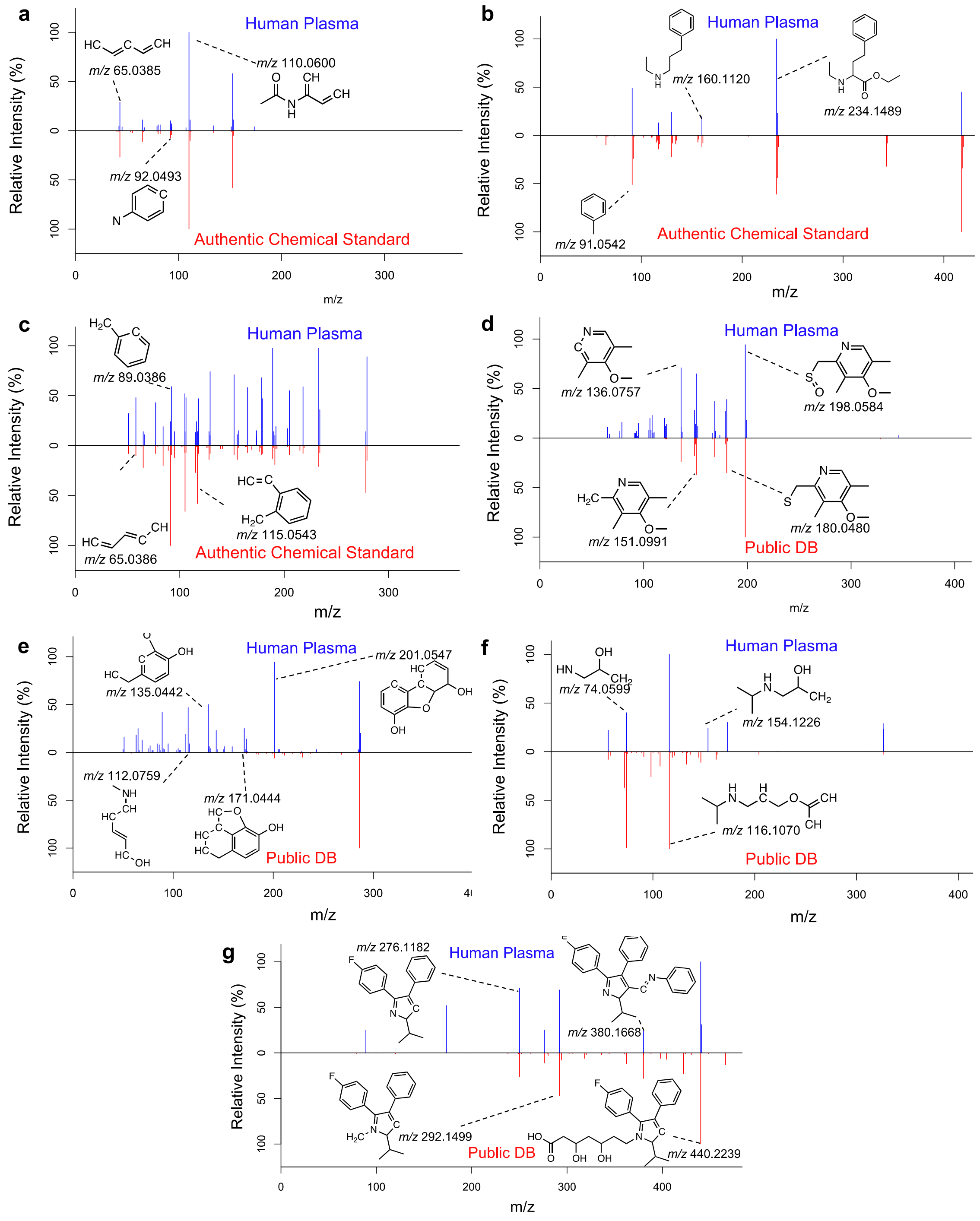

Supplementary Figure 17 Confident annotation of pharmaceuticals detected in human plasma. *Comparison of measured MS*^{2} *fragmentation spectra for* ***a*** *Paracetamol (acetaminophen),* ***b*** *ramipril, and* ***c*** *amitriptyline in human plasma (top) vs authentic chemical standards (bottom) and the corresponding MetFrag-annotated structures of major peaks. Comparison of MS*^{2} *fragmentation spectra for* ***d*** *omeprazole,* ***e*** *morphine,* ***f*** *bisoprolol and* ***g*** *atorvastatin in human plasma (top) vs spectrum from a public database (MassBank of North America; accession codes: MoNA037611, LU138502, EA274410 and EQ301303 for omeprazole, atorvastatin, morphine and bisoprolol, respectively) (bottom) and the corresponding MetFrag-annotated structures of major peaks.*

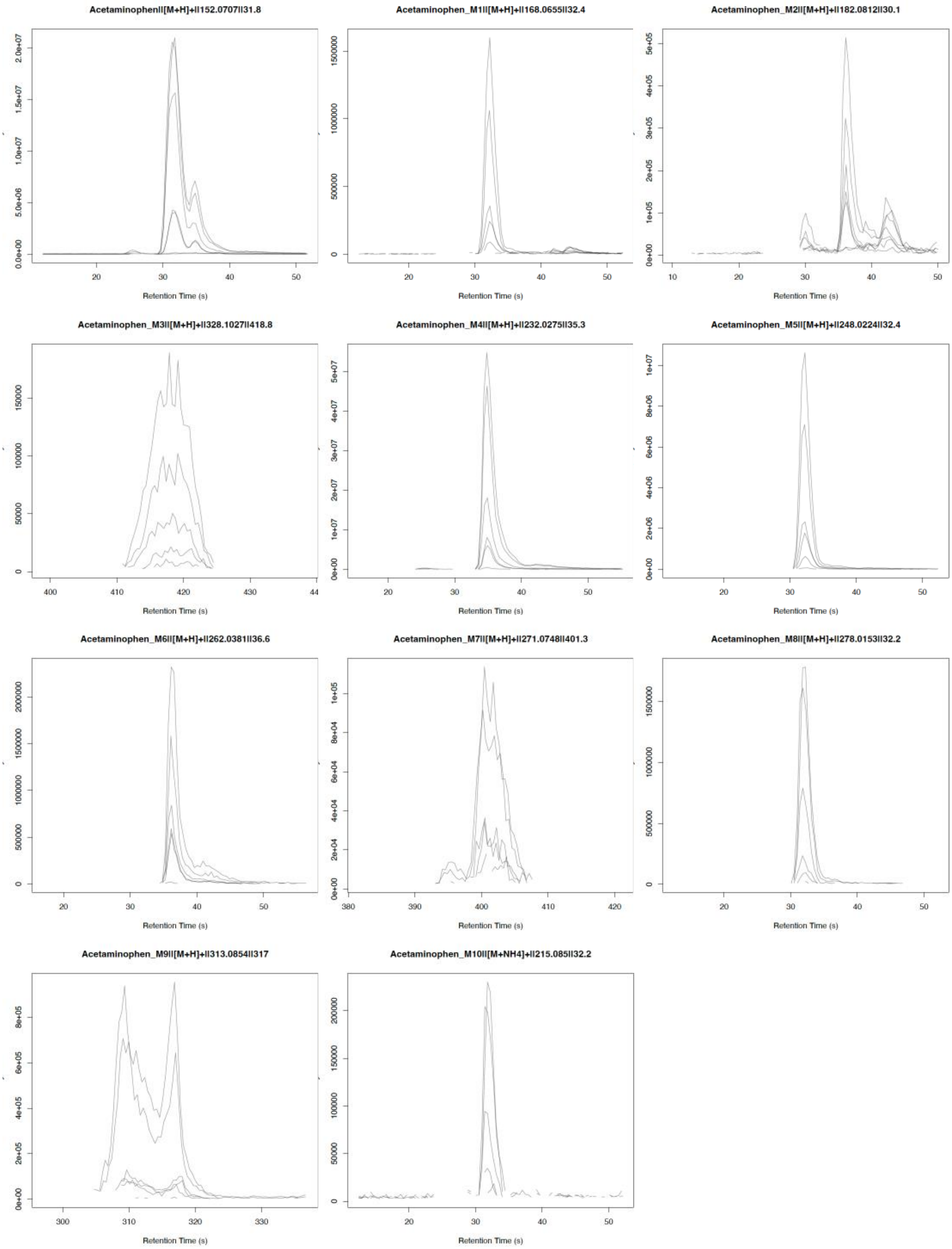
Acetaminophen||[M+H]+||152.0707||31.8
Acetaminophen_M1||[M+H]+||168.0655||32.4
Acetaminophen_M2||[M+H]+||182.0812||30.1
Acetaminophen_M3||[M+H]+||328.1027||418.8
Acetaminophen_M4||[M+H]+||232.0275||35.3
Acetaminophen_M5||[M+H]+||248.0224||32.4
Acetaminophen_M6||[M+H]+||262.0381||36.6
Acetaminophen_M7||[M+H]+||271.0748||401.3
Acetaminophen_M8||[M+H]+||278.0153||32.2
Acetaminophen_M9||[M+H]+||313.0854||317
Acetaminophen_M10||[M+NH4]+||215.085||32.2
Retention Time (s)

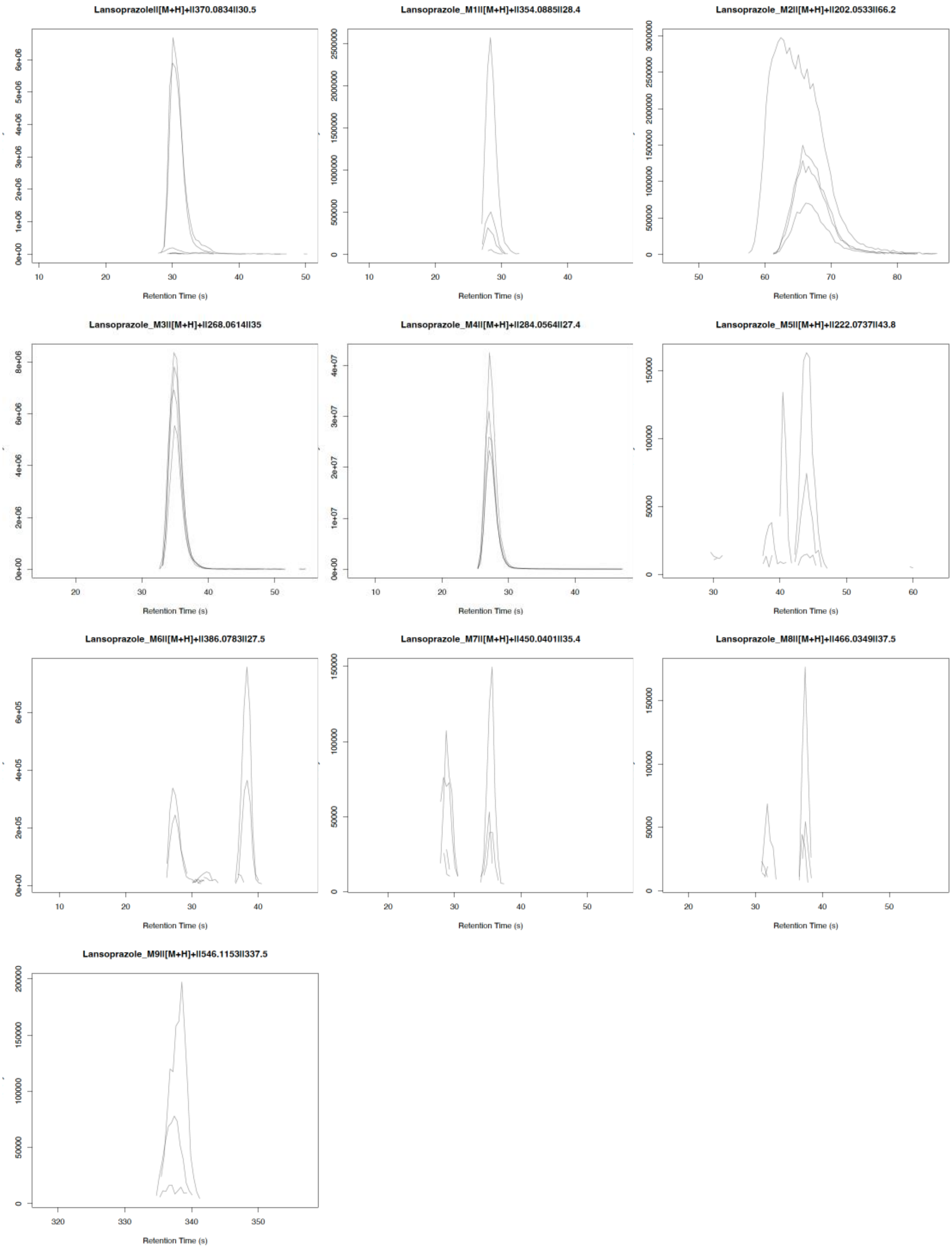
Lansoprazole||[M+H]+||370.0834||30.5
Lansoprazole_M1||[M+H]+||354.0885||28.4
Lansoprazole_M2||[M+H]+||202.0533||66.2
Lansoprazole_M3||[M+H]+||268.0614||35
Lansoprazole_M4||[M+H]+||284.0564||27.4
Lansoprazole_M5||[M+H]+||222.0737||43.8
Lansoprazole_M6||[M+H]+||386.0783||27.5
Lansoprazole_M7||[M+H]+||450.0401||35.4
Lansoprazole_M8||[M+H]+||466.0349||37.5
Lansoprazole_M9||[M+H]+||546.1153||337.5
Retention Time (s)

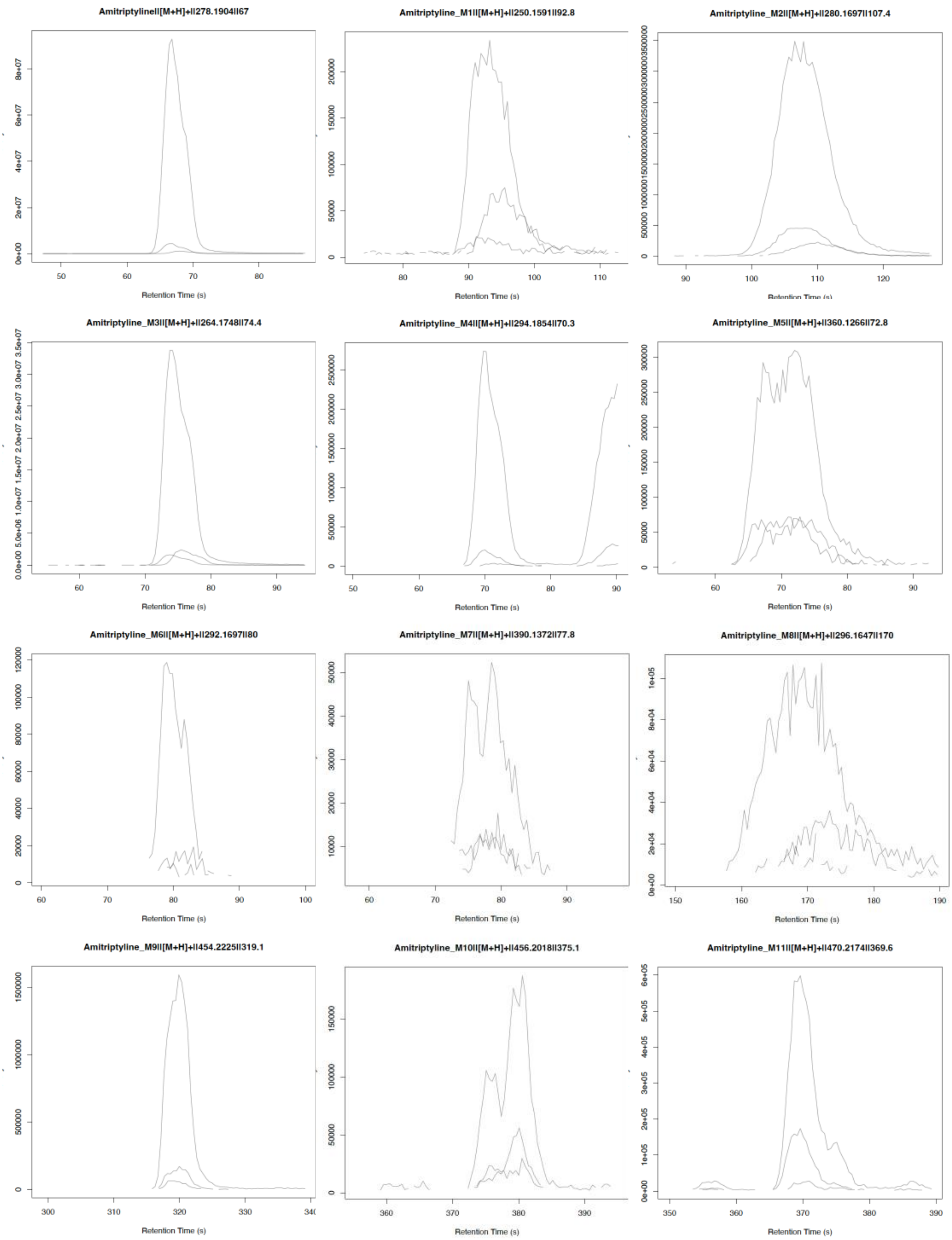
Amitriptyline||[M+H]+||278.1904||67
Amitriptyline_M1||[M+H]+||250.1591||92.8
Amitriptyline_M2||[M+H]+||280.1697||107.4
Amitriptyline_M3||[M+H]+||264.1748||74.4
Amitriptyline_M4||[M+H]+||294.1854||70.3
Amitriptyline_M5||[M+H]+||360.1266||72.8
Amitriptyline_M6||[M+H]+||292.1697||80
Amitriptyline_M7||[M+H]+||390.1372||77.8
Amitriptyline_M8||[M+H]+||296.1647||170
Amitriptyline_M9||[M+H]+||454.2225||319.1
Amitriptyline_M10||[M+H]+||456.2018||375.1
Amitriptyline_M11||[M+H]+||470.2174||369.6
Retention Time (s)

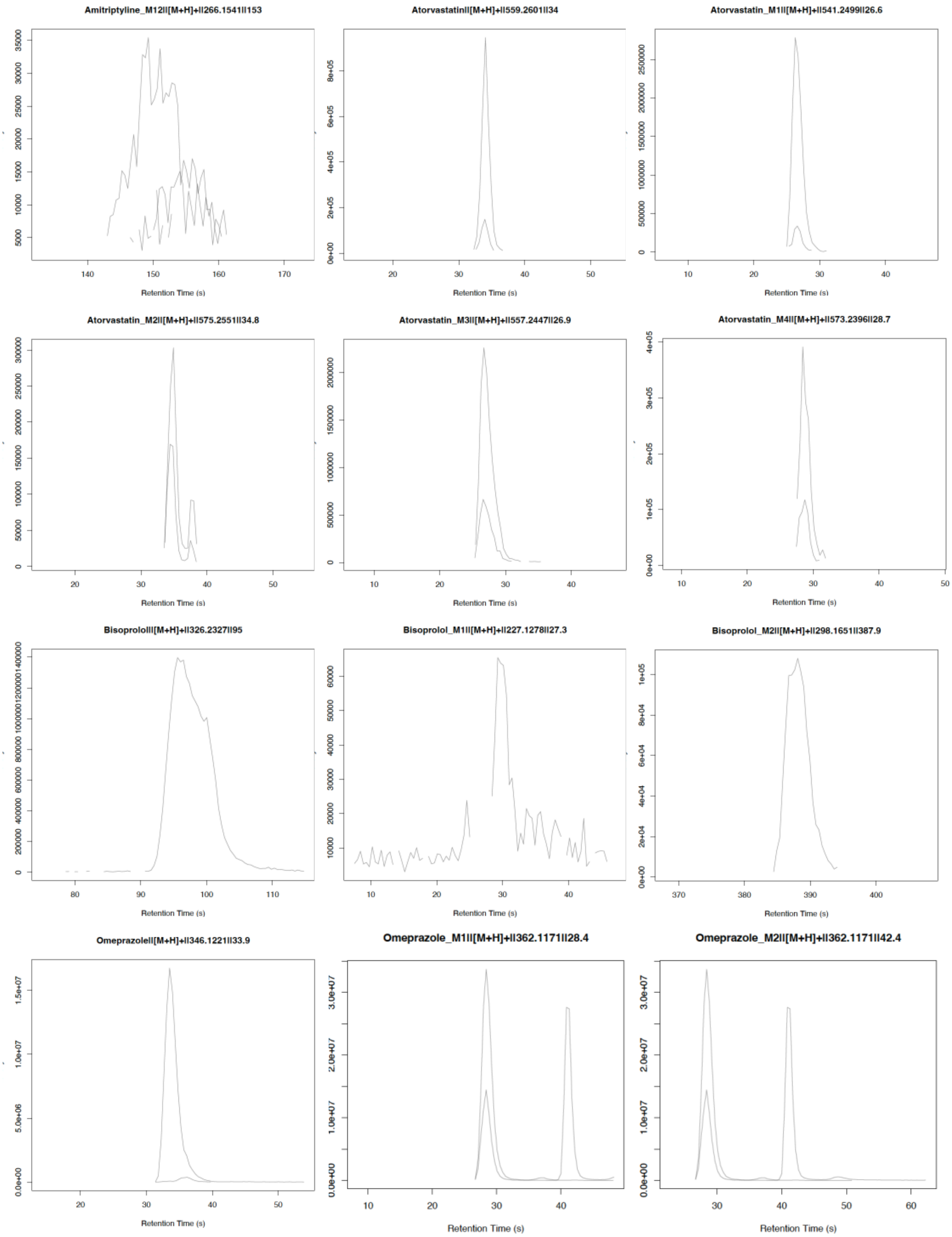
Amitriptyline_M12||[M+H]+||266.1541||153
Atorvastatin||[M+H]+||559.2601||34
Atorvastatin_M1||[M+H]+||541.2499||26.6
Atorvastatin_M2||[M+H]+||575.2551||34.8
Atorvastatin_M3||[M+H]+||557.2447||26.9
Atorvastatin_M4||[M+H]+||573.2396||28.7
Bisoprolol||[M+H]+||326.2327||95
Bisoprolol_M1||[M+H]+||227.1278||27.3
Bisoprolol_M2||[M+H]+||298.1651||387.9
Omeprazole||[M+H]+||346.1221||33.9
Omeprazole_M1||[M+H]+||362.1171||28.4
Omeprazole_M2||[M+H]+||362.1171||42.4
Retention Time (s)

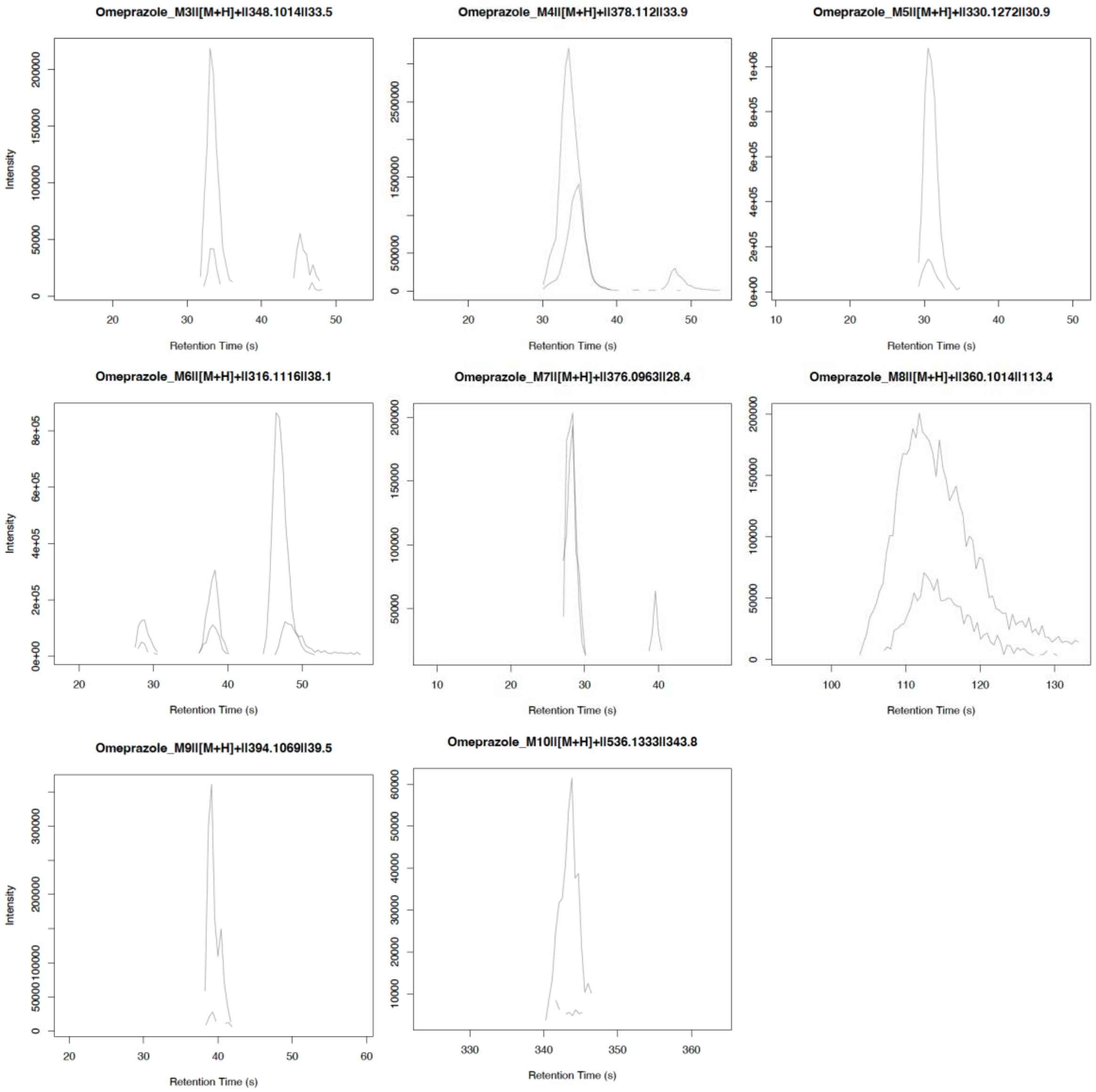

Supplementary Figure 18 Chromatographic peaks of pharmaceuticals and their biotransformation products measured in human plasma. *Extracted ion chromatograms (EICs) of Paracetamol (acetaminophen), lansoprazole, amitriptyline, atorvastatin, bisoprolol and omeprazole, and their biotransformation products measured in the human plasma samples by HILIC positive UHPLC-MS untargeted metabolomics.*

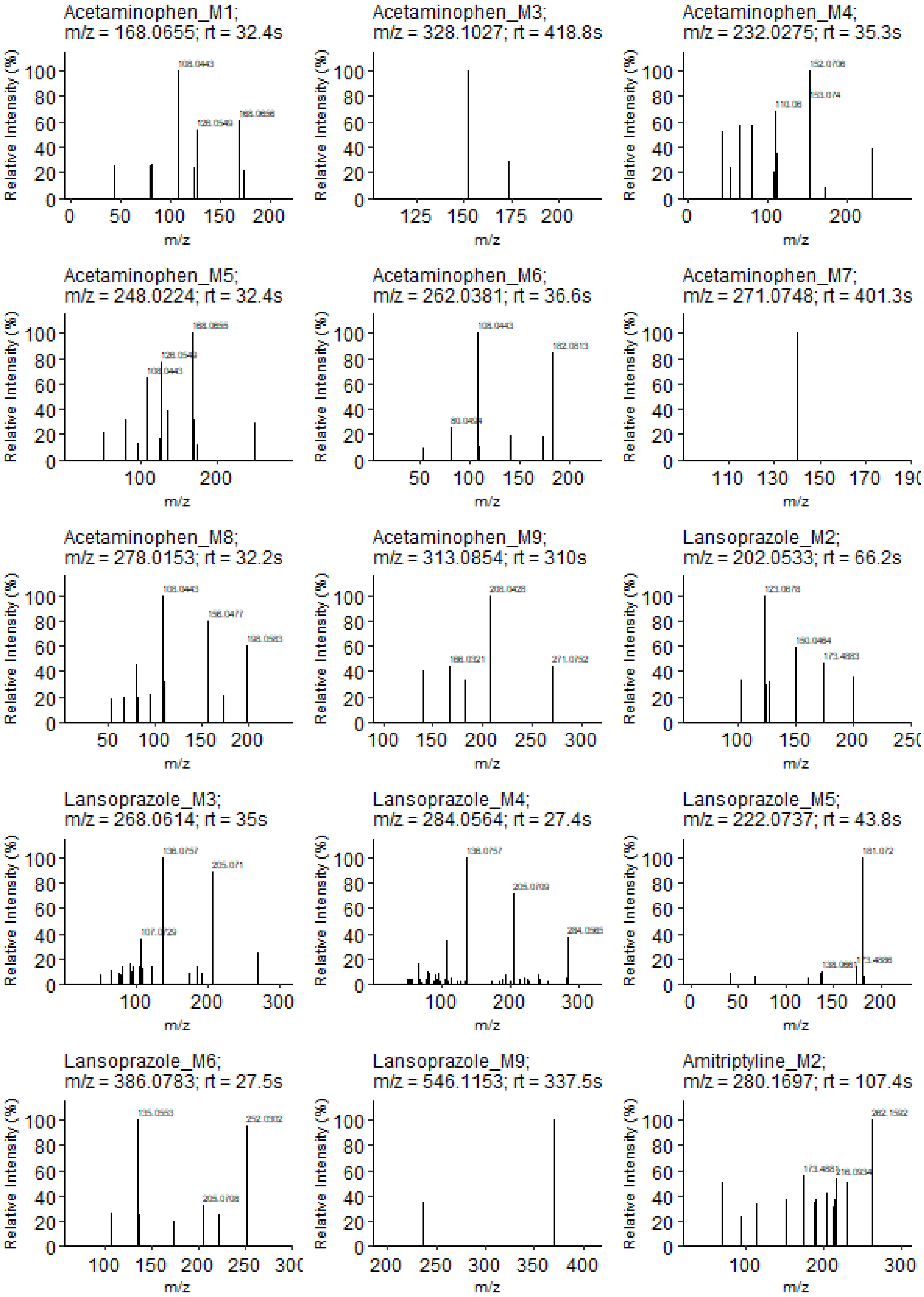
Acetaminophen_M1; m/z = 168.0655; rt = 32.4s
Acetaminophen_M3; m/z = 328.1027; rt = 418.8s
Acetaminophen_M4; m/z = 232.0275; rt = 35.3s
Acetaminophen_M5; m/z = 248.0224; rt = 32.4s
Acetaminophen_M6; m/z = 262.0381; rt = 36.6s
Acetaminophen_M7; m/z = 271.0748; rt = 401.3s
Acetaminophen_M8; m/z = 278.0153; rt = 32.2s
Acetaminophen_M9; m/z = 313.0854; rt = 310s
Lansoprazole_M2; m/z = 202.0533; rt = 66.2s
Lansoprazole_M3; m/z = 268.0614; rt = 35s
Lansoprazole_M4; m/z = 284.0564; rt = 27.4s
Lansoprazole_M5; m/z = 222.0737; rt = 43.8s
Lansoprazole_M6; m/z = 386.0783; rt = 27.5s
Lansoprazole_M9; m/z = 546.1153; rt = 337.5s
Amitriptyline_M2; m/z = 280.1697; rt = 107.4s
Relative Intensity (%)
m/z

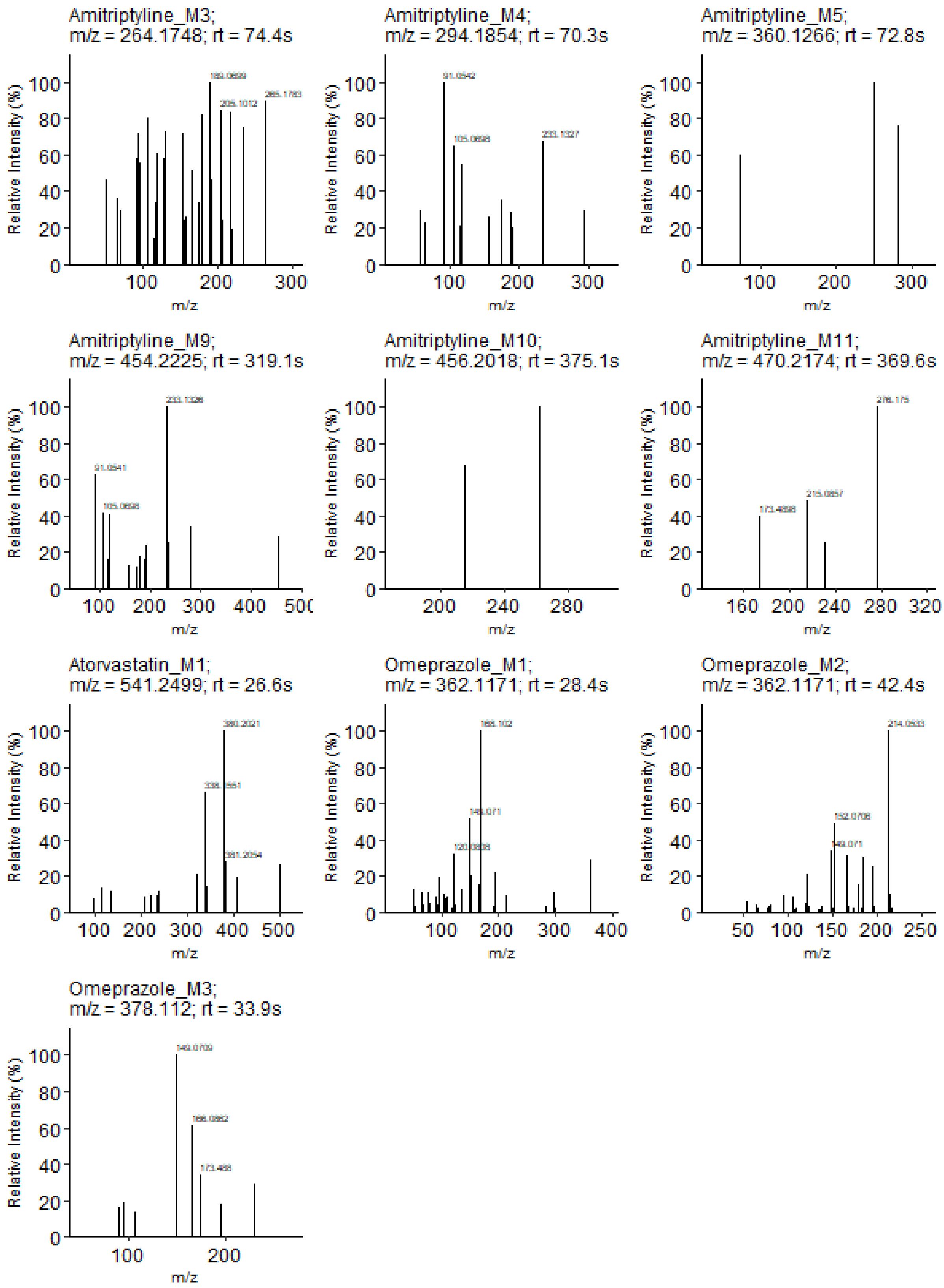

Supplementary Figure 19 Mass spectra of pharmaceuticals and their biotransformation products detected in human plasma. *MS^2 fragmentation spectra of the biotransformation products of Paracetamol (acetaminophen), amitriptyline, omeprazole, atorvastatin and bisoprolol measured in human plasma samples by HILIC positive UHPLC-MS untargeted metabolomics.*

Supplementary Table 11 Implementing the untargeted ADME/TK workflow on human plasma UHPLC-MS untargeted metabolomics data. *The number of samples defined as either 'exposed' or 'control' with numbers disaggregated for self-reported gender, either male (M) or female (F), in brackets, number of putative xenobiotic-related features remaining after implementing the 3 intensity-based filters, and the number of annotated biotransformation products annotated to either MSI Level 3 (MS^1 match only) or MSI Level 2 (supporting MS^2 spectrum) are reported for each xenobiotic confidently detected in the dataset.*

Pharmaceutical	Number of 'exposed' samples	Number of 'control' samples	Number of putative xenobiotic-related features	Number of molecular formulae annotated biotransformation products (MS^1 only)	Number of structurally annotated biotransformation products (MetFrag)
Paracetamol (Acetaminophen)	7 (5F;2M)	14 (13F; 1M)	70	2	8
Omeprazole	3 (3F)	18 (15F; 3M)	89	7	3
Atorvastatin	2 (2M)	19 (18F; 1M)	201	3	1
Ramipril	0	21 (18F; 3M)	0	N/A	N/A
Lansoprazole	6 (4F; 2M)	15 (14F; 1M)	74	3	6
Bisoprolol	1 (1M)	20 (18F; 2M)	73	2	0
Amitriptyline	3 (3F)	18 (15F; 3M)	51	5	7
Morphine	0	21 (18F; 3M)	0	N/A	N/A

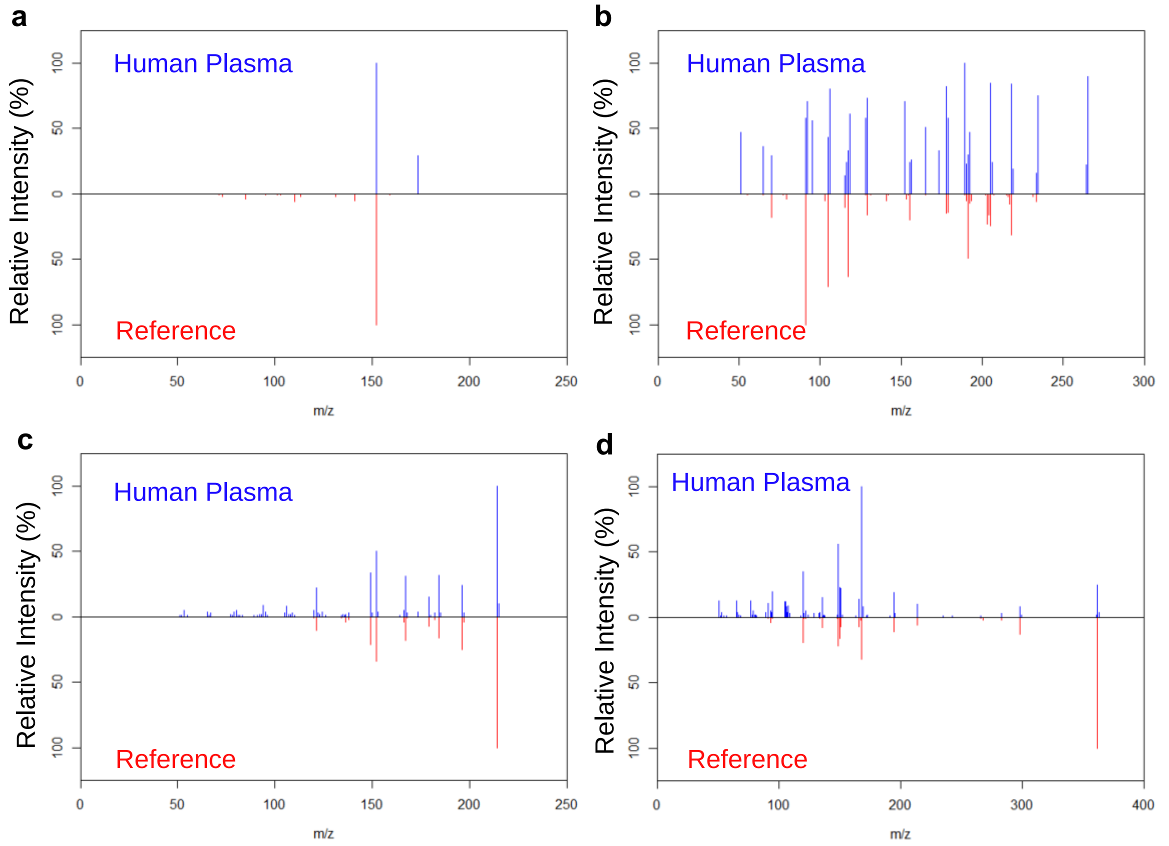

Supplementary Figure 20 Confident annotation of four biotransformation products detected in human plasma. *Comparison of measured MS² fragmentation spectra for **a** acetaminophen glucuronide (acetaminophen M3), **b** nortriptyline (amitriptyline M3), and **c** 5-hydroxyomeprazole (omeprazole M2) and **d** omeprazole sulfone (omeprazole M1) in human plasma (top) vs reference spectra (bottom) from a public database (**a** and **b**: MassBank of North America, accession codes: FiehnHILIC000149 and EQ369204, respectively; **c** and **d**: mzCloud, accession codes: Reference1851 and Reference2488, respectively).*

Supplementary References

1 Ridder, L. & Wagener, M. SyGMa: combining expert knowledge and empirical scoring in the prediction of metabolites. *ChemMedChem* **3**, 821-832, doi:10.1002/cmdc.200700312 (2008).

2 Speed, B. *et al.* Pharmacokinetics, distribution, and metabolism of [14C]sunitinib in rats, monkeys, and humans. *Drug Metab Dispos* **40**, 539-555, doi:10.1124/dmd.111.042853 (2012).

3 Ruttkies, C., Schymanski, E. L., Wolf, S., Hollender, J. & Neumann, S. MetFrag relaunched: incorporating strategies beyond in silico fragmentation. *J Cheminform* **8**, 3, doi:10.1186/s13321-016-0115-9 (2016).

4 Sumner, L. W. *et al.* Proposed minimum reporting standards for chemical analysis. *Metabolomics* **3**, 211-221, doi:10.1007/s11306-007-0082-2 (2007).

5 Schymanski, E. L. *et al.* Identifying Small Molecules via High Resolution Mass Spectrometry: Communicating Confidence. *Environmental Science & Technology* **48**, 2097-2098, doi:10.1021/es5002105 (2014).
