# Supplementary material for: Simultaneously discovering the fate and biochemical effects of pharmaceuticals through untargeted metabolomics
Source: Nat Commun. 2023 Aug 3;14:4653. doi: 10.1038/s41467-023-40333-7 (PMC10400635; doi:10.1038/s41467-023-40333-7)
Supplement: Supplementary file 3 — Description of Additional Supplementary Files [file 41467_2023_40333_MOESM3_ESM.pdf]

Description of Additional Supplementary Files

File Name: Supplementary Data 1
Description: List of biotransformation products of sunitinib and KU60648 predicted by "Generate Expected Compounds" tool of Compound Discoverer (Thermo Scientific) and SyGMa[1]. The molecular formula and exact mass for each predicted biotransformation product are reported. The SyGMa score is also reported for SyGMA predictions.

File Name: Supplementary Data 2
Description: Biotransformation products of sunitinib detected in the plasma and cardiac tissue of rats treated with sunitinib. Each biotransformation product is described by its molecular formulae and the type of biotransformation step (phase I or phase II) from parent which leads to its production. The measured *m/z*, retention time (RT) and ion form of a representative feature are reported for each UHPLC-MS assay in which a given compound was detected. Where corresponding MS/MS fragmentation data was acquired for a given feature, the Metabolomics Standards Initiative (MSI) and Schymanski annotation confidence levels and a list of fragment ions annotated with molecular formulae by MetFrag (MetFrag Explained MS/MS Peaks) are reported. Also reported is the correlation coefficient (R) and corresponding Holm's adjusted *p*-value calculated by Pearson's-based correlation analysis between the peak intensities of each given feature and the peak intensities of the selected representative feature of sunitinib (HILIC positive, *m/z* 399.2185, rt 147.4s) measured in the plasma samples of treated rats.

File Name: Supplementary Data 3
Description: Biotransformation products of KU60648 detected in the plasma and cardiac tissue of rats treated with KU60648. Each biotransformation product is described by its molecular formulae and the type of biotransformation step (phase I or phase II) from parent which leads to its production. The measured *m/z*, retention time (RT) and ion form of a representative feature are reported for each UHPLC-MS assay in which a given compound was detected. Where corresponding MS/MS fragmentation data was acquired for a given feature, the Metabolomics Standards Initiative (MSI) and Schymanski annotation confidence levels and a list of fragment ions annotated with molecular formulae by MetFrag (MetFrag Explained MS/MS Peaks) are reported. Also reported is the correlation coefficient (R) and corresponding Holm's adjusted *p*-value calculated by Pearson's-based correlation analysis between the peak intensities of each given feature and the peak intensities of the selected representative feature of KU60648 (HILIC positive, *m/z* 583.2373, rt 277.3s) measured in the plasma samples of treated rats.

File Name: Supplementary Data 4
Description: Polar metabolites and lipids (annotated to MSI level 2) found to be significantly altered in plasma of rats exposed to sunitinib compared to biological controls. The *m/z*, retention time (RT) and ion form of features corresponding to each polar metabolite or lipid species the quality of lipid species annotation (LipidSearch Annotation Grade), where applicable, and the ratio of the feature's median intensity in samples from rats exposed to sunitinib relative to biological control samples at the specified time point is reported (blue shading indicates increase in exposed samples, red shading indicates decrease in exposed samples, relative to biological controls). Differences between exposed and biological control samples were evaluated using the two-tailed student's *t*-test with FDR correction (*q*-value) (N = 5).

File Name: Supplementary Data 5
Description: Lipids (annotated to MSI level 2) found to be significantly altered in cardiac tissue of rats exposed to sunitinib compared to biological controls. The *m/z*, retention time (RT) and ion form of features corresponding to each lipid species, the quality of lipid species annotation (LipidSearch Annotation Grade), and the ratio of the feature's median intensity in samples from rats exposed to sunitinib relative to biological control samples is reported (blue shading indicates increase in exposed samples, red shading indicates decrease in exposed samples, relative to biological controls). Differences between exposed and biological control samples were evaluated using the two-tailed student's *t*-test with FDR correction (*q*-value) (N = 5).

File Name: Supplementary Data 6
Description: Lipids (annotated to MSI level 2) significantly correlated to sunitinib in cardiac tissue of rats exposed to sunitinib. The *m/z*, retention time (RT) and ion form of features corresponding to each

lipid species, the quality of lipid species annotation (LipidSearch Annotation Grade), and the Spearman's correlation coefficient (ρ) (blue shading indicates positive correlation; red indicates negative correlation) and corresponding p-value for the correlation between feature intensity and sunitinib across cardiac tissue samples from rats exposed to sunitinib is reported for lipids that are significantly correlated (p-value < 0.05) (N = 5).

File Name: Supplementary Data 7
Description: Polar metabolites and lipids (annotated to MSI level 2) found to be significantly altered in plasma of rats exposed to KU60648 compared to biological controls. The *m/z*, retention time (RT) and ion form of features corresponding to each polar metabolite or lipid species, the quality of lipid species annotation (LipidSearch Annotation Grade), where applicable, and the ratio of the feature's median intensity in samples from rats exposed to KU60648 relative to biological control samples at the specified time point is reported (blue shading indicates increase in exposed samples, red shading indicates decrease in exposed samples, relative to biological controls). Differences between exposed and biological control samples were evaluated using the two-tailed student's t-test with FDR correction (q-value) (N = 5).

File Name: Supplementary Data 8
Description: Lipids (annotated to MSI level 2) found to be significantly altered in cardiac tissue of rats exposed to KU60648 compared to biological controls. The *m/z*, retention time (RT) and ion form of features corresponding to each lipid species, the quality of lipid species annotation (LipidSearch Annotation Grade), and the ratio of the features median intensity in samples from rats exposed to KU60648 against the features median intensity in biological control samples is reported (blue shading indicates increase in exposed samples, red shading indicates decrease in exposed samples, relative to biological controls). Differences between exposed and biological control samples were evaluated using the two-tailed student's t-test with FDR correction (q-value) (N = 5).

File Name: Supplementary Data 9
Description: Lipids (annotated to MSI level 2) significantly correlated to KU60648 in cardiac tissue of rats exposed to KU60648. The *m/z*, retention time (RT) and ion form of features corresponding to each lipid species, the quality of lipid species annotation (LipidSearch Annotation Grade), and the Spearman's correlation coefficient (ρ) (blue shading indicates positive correlation; red shading indicates negative correlation) and corresponding p-value for the correlation between feature intensity and KU60648 across cardiac tissue samples from rats exposed to KU60648 (N = 5) is reported for lipids that are significantly correlated (p-value < 0.05).

File Name: Supplementary Data 10
Description: Biotransformation products of sunitinib detected in the intracellular extracts and extracellular culture medium of hiPSC-cardiomyocyte cultures exposed to sunitinib. Each biotransformation product is described by its molecular formulae and the type of biotransformation step (phase I or phase II) from parent which leads to its production. The measured *m/z*, retention time (RT) and ion form of a representative feature are reported for each UHPLC-MS assay in which a given compound was detected. Where corresponding MS/MS fragmentation data was acquired for a given feature, the Metabolomics Standards Initiative (MSI) and Schymanski annotation confidence levels and a list of fragment ions annotated with molecular formulae by MetFrag (MetFrag Explained MS/MS Peaks) are reported.

File Name: Supplementary Data 11
Description: Biotransformation products of Paracetamol (acetaminophen) detected in human plasma samples. Each biotransformation product is described by its molecular formulae and the type of biotransformation step (phase I or phase II) from parent which leads to its production. The measured *m/z*, retention time (RT) and ion form of a representative feature are reported for each UHPLC-MS assay in which a given compound was detected. Where corresponding MS/MS fragmentation data was acquired for a given feature, the Metabolomics Standards Initiative (MSI) and Schymanski annotation confidence levels and a list of fragment ions annotated with molecular formulae by MetFrag (MetFrag Explained MS/MS Peaks) are reported.

File Name: Supplementary Data 12
Description: Biotransformation products of lansoprazole detected in human plasma samples. Each biotransformation product is described by its molecular formulae and the type of biotransformation

step (phase I or phase II) from parent which leads to its production. The measured *m/z*, retention time (RT) and ion form of a representative feature are reported for each UHPLC-MS assay in which a given compound was detected. Where corresponding MS/MS fragmentation data was acquired for a given feature, the Metabolomics Standards Initiative (MSI) and Schymanski annotation confidence levels and a list of fragment ions annotated with molecular formulae by MetFrag (MetFrag Explained MS/MS Peaks) are reported.

File Name: Supplementary Data 13
Description: Biotransformation products of amitriptyline detected in human plasma samples. Each biotransformation product is described by its molecular formulae and the type of biotransformation step (phase I or phase II) from parent which leads to its production. The measured *m/z*, retention time (RT) and ion form of a representative feature are reported for each UHPLC-MS assay in which a given compound was detected. Where corresponding MS/MS fragmentation data was acquired for a given feature, the Metabolomics Standards Initiative (MSI) and Schymanski annotation confidence levels and a list of fragment ions annotated with molecular formulae by MetFrag (MetFrag Explained MS/MS Peaks) are reported.

File Name: Supplementary Data 14
Description: Biotransformation products of atorvastatin detected in human plasma samples. Each biotransformation product is described by its molecular formulae and the type of biotransformation step (phase I or phase II) from parent which leads to its production. The measured *m/z*, retention time (RT) and ion form of a representative feature are reported for each UHPLC-MS assay in which a given compound was detected. Where corresponding MS/MS fragmentation data was acquired for a given feature, the Metabolomics Standards Initiative (MSI) and Schymanski annotation confidence levels and a list of fragment ions annotated with molecular formulae by MetFrag (MetFrag Explained MS/MS Peaks) are reported.

File Name: Supplementary Data 15
Description: Biotransformation products of bisoprolol detected in human plasma samples. Each biotransformation product is described by its molecular formulae and the type of biotransformation step (phase I or phase II) from parent which leads to its production. The measured *m/z*, retention time (RT) and ion form of a representative feature are reported for each UHPLC-MS assay in which a given compound was detected. Where corresponding MS/MS fragmentation data was acquired for a given feature, the Metabolomics Standards Initiative (MSI) and Schymanski annotation confidence levels and a list of fragment ions annotated with molecular formulae by MetFrag (MetFrag Explained MS/MS Peaks) are reported.

File Name: Supplementary Data 16
Description: Biotransformation products of omeprazole detected in human plasma samples. Each biotransformation product is described by its molecular formulae and the type of biotransformation step (phase I or phase II) from parent which leads to its production. The measured *m/z*, retention time (RT) and ion form of a representative feature are reported for each UHPLC-MS assay in which a given compound was detected. Where corresponding MS/MS fragmentation data was acquired for a given feature, the Metabolomics Standards Initiative (MSI) and Schymanski annotation confidence levels and a list of fragment ions annotated with molecular formulae by MetFrag (MetFrag Explained MS/MS Peaks) are reported.
