# Supplementary material for: Simultaneously discovering the fate and biochemical effects of pharmaceuticals through untargeted metabolomics
Source: Nat Commun. 2023 Aug 3;14:4653. doi: 10.1038/s41467-023-40333-7 (PMC10400635; doi:10.1038/s41467-023-40333-7)
Supplement: Supplementary file 20 — Reporting Summary [file 41467_2023_40333_MOESM20_ESM.pdf]

nature portfolio

Corresponding author(s): Mark R. Viant

Last updated by author(s): Jul 10, 2023

Reporting Summary

Nature Portfolio wishes to improve the reproducibility of the work that we publish. This form provides structure for consistency and transparency in reporting. For further information on Nature Portfolio policies, see our Editorial Policies and the Editorial Policy Checklist.

Statistics

For all statistical analyses, confirm that the following items are present in the figure legend, table legend, main text, or Methods section.

n/a	Confirmed	
☐	☒	The exact sample size (n) for each experimental group/condition, given as a discrete number and unit of measurement
☐	☒	A statement on whether measurements were taken from distinct samples or whether the same sample was measured repeatedly
☐	☒	The statistical test(s) used AND whether they are one- or two-sided<br>*Only common tests should be described solely by name; describe more complex techniques in the Methods section.*
☒	☐	A description of all covariates tested
☐	☒	A description of any assumptions or corrections, such as tests of normality and adjustment for multiple comparisons
☐	☒	A full description of the statistical parameters including central tendency (e.g. means) or other basic estimates (e.g. regression coefficient) AND variation (e.g. standard deviation) or associated estimates of uncertainty (e.g. confidence intervals)
☐	☒	For null hypothesis testing, the test statistic (e.g. F, t, r) with confidence intervals, effect sizes, degrees of freedom and P value noted<br>*Give P values as exact values whenever suitable.*
☒	☐	For Bayesian analysis, information on the choice of priors and Markov chain Monte Carlo settings
☒	☐	For hierarchical and complex designs, identification of the appropriate level for tests and full reporting of outcomes
☐	☒	Estimates of effect sizes (e.g. Cohen's d, Pearson's r), indicating how they were calculated

Our web collection on statistics for biologists contains articles on many of the points above.

Software and code

Policy information about availability of computer code

Data collection	Thermo XCalibur software (Thermo Fisher Scientific)
Data analysis	Proteowizard v3.0; XCMS v1.46.0 (run in the Galaxy environment); R programming language; msPurity v1.16 (https://www.bioconductor.org/packages/release/bioc/html/msPurity.html); BEAMSpy (v1.0); Compound Discoverer v3.0 (Thermo Fisher Scientific); SyGMa v1.1; MetFrag; MetabolAnalyze v1.3; pmp v1.1 (https://bioconductor.org/packages/release/bioc/html/pmp.html); structToolbox v1.6.0 & v1.6.1 (https://bioconductor.org/packages/release/bioc/html/structToolbox.html); LipidSearch v4.2 (Thermo Fisher Scientific); RcmdrMisc v2.7 (https://cran.r-project.org/web/packages/RcmdrMisc/index.html); Cytoscape v3.7.2

For manuscripts utilizing custom algorithms or software that are central to the research but not yet described in published literature, software must be made available to editors and reviewers. We strongly encourage code deposition in a community repository (e.g. GitHub). See the Nature Portfolio guidelines for submitting code & software for further information.

Data

Policy information about availability of data

All manuscripts must include a data availability statement. This statement should provide the following information, where applicable:

- Accession codes, unique identifiers, or web links for publicly available datasets
- A description of any restrictions on data availability
- For clinical datasets or third party data, please ensure that the statement adheres to our policy

Untargeted UHPLC-MS(/MS) metabolomics raw and derived data and associated metadata that support the findings of this study have been deposited in

MetaboLights with the accession code MTBLS2746 (https://www.ebi.ac.uk/metabolights/MTBLS2746). Additionally, fragmentation data (MS/MS) used to support findings presented in the manuscript has been deposited in MassBank (https://massbank.eu/MassBank/; accession codes: MSBNK-UoB-XB000xxx, where xxx is 101-112, 200-215, 300-306, 400-406, 500-504, 600, 700-701, 800, or 900-902).

Research involving human participants, their data, or biological material

Policy information about studies with human participants or human data. See also policy information about sex, gender (identity/presentation), and sexual orientation and race, ethnicity and racism.

Reporting on sex and gender	Gender was recorded in the study, based on self-reporting, and the number of female and male subjects is presented in the manuscript for completeness. However, gender was not considered in the study design and no gender analysis was conducted as we were not seeking male- or female-specific evidence of drug metabolism.
Reporting on race, ethnicity, or other socially relevant groupings	No socially constructed or socially relevant categorical variables were considered in this study.
Population characteristics	The 21 human participants were age 24-62, and reported to have taken some common medicine in the 24-hours prior to blood donation.
Recruitment	Wider recruitment of human research particpants was orginally conducted by the University of Birmingham's Human Biomaterials Resource Centre (HBRC), with the 21 volunteers is this study selected based on metadata that indicated that they were taking at least one type of medication. All donors gave informed consent. They were not compensated.
Ethics oversight	Samples were obtained from the University of Birmingham's Human Biomaterials Resource Centre (HBRC), which holds ethical approval from an NHS Research Ethics Committee (NRES Committee North West – Haydock; Ref 20/NW/0001) to provide human biomaterials and associated data for a broad spectrum of biomedical research. Human biomaterials and associated data were obtained in accordance with the Human Tissue Act 2004 and associated Codes of Practice, and project specific use of human biomaterials and associated data were subject to the HBRC Access Review panel for ethical approval and sponsorship under the UK Policy Framework for Health and Social Care Research.

Note that full information on the approval of the study protocol must also be provided in the manuscript.

Field-specific reporting

Please select the one below that is the best fit for your research. If you are not sure, read the appropriate sections before making your selection.

[x] Life sciences [ ] Behavioural & social sciences [ ] Ecological, evolutionary & environmental sciences

For a reference copy of the document with all sections, see nature.com/documents/nr-reporting-summary-flat.pdf

Life sciences study design

All studies must disclose on these points even when the disclosure is negative.

Sample size	Based on the exploratory nature of studies presented in this manuscript, formal power analyses would be inappropriate. Regarding in vivo studies, based on the known variance in toxicokinetics following oral dosing, N=5 individual animals was deemed sufficient (this is in line with best practice guidance from LASA/NC3Rs). Biological variation in endogenous metabolites and lipids for all studies was demonstrated to be acceptable as evidenced by median relative standard deviations < 35%. Effect size (metabolome and lipidome) was large in the presence of the tested compounds.
Data exclusions	No data were excluded from analyses.
Replication	Animal exposure studies used at least N=5 replicates (separate animals) within the toxicology study as indicated above. The in vivo exposure study to measure the drug's metabolism repeated an earlier rat study used by the authors, measuring histopathological endpoints.<br>The cardiomyocyte exposure study to measure the drug's metabolism used N=3 replicates, each formed from cardiomyocytes from separate vials. This was a repeat of the design previously used by the authors to measure high content biology (imaging) endpoints. Agreement with previously published biotransformation products was high. On this basis, no further replication of the cardiomyocyte exposures was deemed necessary. In all cases, measurement of reported xenobiotics and biotransformation products were replicated across 80% of exposed samples.
Randomization	For rat exposure experiments, animals were grouped randomly: groups 1 – 4 were formed of N = 5 male rats for KU60648 exposure study (N=10 total treated with KU60648, N=10 total vehicle controls), groups 5 – 8 were formed of N = 5 female rats and used for sunitinib exposure studies (N=10 total treated with sunitinib, N=10 total vehicle controls).<br><br>All biological samples were (block) randomised into extraction batches for sample preparation, and re-(block)randomised into analytical batches for sample analysis.
Blinding	No blinding was conducted. The rat and cardiomyocyte studies used defined treatment groups and this sample class information was required for the data analysis approaches applied, i.e., to extract xenobiotic-related features measured in treated samples and not control samples. All 21 healthy human volunteers formed one group. There were no descriptors that could have been blinded for this study.

Reporting for specific materials, systems and methods

We require information from authors about some types of materials, experimental systems and methods used in many studies. Here, indicate whether each material, system or method listed is relevant to your study. If you are not sure if a list item applies to your research, read the appropriate section before selecting a response.

Materials & experimental systems

n/a	Involved in the study
☒	☐ Antibodies
☒	☐ Eukaryotic cell lines
☒	☐ Palaeontology and archaeology
☐	☒ Animals and other organisms
☒	☐ Clinical data
☒	☐ Dual use research of concern
☒	☐ Plants

Methods

n/a	Involved in the study
☒	☐ ChIP-seq
☒	☐ Flow cytometry
☒	☐ MRI-based neuroimaging

Animals and other research organisms

Policy information about studies involving animals; ARRIVE guidelines recommended for reporting animal research, and Sex and Gender in Research

Laboratory animals	Han-Wistar - Crl:WI(Han). Weight: 240 - 260g or 220 - 240g for male and female, respectively. Age approximately 7 weeks.
Wild animals	The study did not involve wild animals.
Reporting on sex	Sunitinib exposure studies and matching controls were performed in female rats only (total: 20). KU60648 exposures and matching controls were performed in male rats only (total: 20). This study design was selected based on prior measured data describing pharmacokinetic and toxicodynamic behaviour of sunitinib and KU60648 in rats of both sexes. Analyses compared exposed to matched controls (i.e., samples from same sex animals).
Field-collected samples	The study did not involve samples collected from the field.
Ethics oversight	Studies were performed in accordance with the United Kingdom Animal (Scientific Procedures) Act 1986, were subject to local ethics committee (AstraZeneca Animal Welfare Review Board and Babraham Institute Animal Welfare and Ethical Review Board) approval and in line with project and personal license conditions.

Note that full information on the approval of the study protocol must also be provided in the manuscript.
